# Building the Leviathan – Voluntary centralisation of punishment power sustains cooperation in humans

**DOI:** 10.1038/srep20767

**Published:** 2016-02-18

**Authors:** Jörg Gross, Zsombor Z. Méder, Sanae Okamoto-Barth, Arno Riedl

**Affiliations:** 1 Department of Cognitive Neuroscience, Faculty of Psychology and Neuroscience, Maastricht University, P.O. Box 616, 6200 MD Maastricht, The Netherlands; 2 Department of Economics (AE1), School of Business and Economics, Maastricht University, P.O. Box 616, 6200 MD Maastricht, The Netherlands; 3 Humanities, Arts and Social Sciences, Singapore University of Technology and Design, 487372, Singapore

## Abstract

The prevalence of cooperation among humans is puzzling because cooperators can be exploited by free riders. Peer punishment has been suggested as a solution to this puzzle, but cumulating evidence questions its robustness in sustaining cooperation. Amongst others, punishment fails when it is not powerful enough, or when it elicits counter-punishment. Existing research, however, has ignored that the distribution of punishment power can be the result of social interactions. We introduce a novel experiment in which individuals can transfer punishment power to others. We find that while decentralised peer punishment fails to overcome free riding, the voluntary transfer of punishment power enables groups to sustain cooperation. This is achieved by non-punishing cooperators empowering those who are willing to punish in the interest of the group. Our results show how voluntary power centralisation can efficiently sustain cooperation, which could explain why hierarchical power structures are widespread among animals and humans.

The scale of cooperation observed among humans remains a puzzle for the social and biological sciences. Cooperative efforts bear the risk of exploitation by selfish agents who can reap the benefits without themselves contributing to the common good. Nevertheless, sustained cooperation is frequently observed in human societies^1^,^2^,^3^,^4^. Peer punishment has been proposed as a possible solution to overcome this free rider problem^5^,^6^,^7^,^8^,^9^,^10^,^11^,^12^,^13^. Experiments on public goods dilemmas showed that cooperation deteriorates quickly in the absence of sanctioning mechanisms, but can stabilize when peer punishment of free riders is possible^6^,^9^,^10^,^14^,^15^.

However, accumulated evidence documents crucial limitations of peer punishment in its ability to sustain cooperation and foster welfare. First, punishment power needs to be sufficiently high, meaning that the cost of punishment for the punisher has to be sufficiently low relative to its effect on the punished^13^,^16^,^17^,^18^,^19^,^20^. Second, non-cooperators sometimes punish cooperators out of spite or retribution, thereby undermining cooperation^21^,^22^,^23^,^24^,^25^,^26^,^27^,^28^. Further, excessive use of punishment can stabilize cooperation but at the cost of reduced group welfare^15^,^16^,^21^,^29^,^30^,^31^,^32^. Finally, not all members of a group participate in the punishment of non-cooperators. It is frequently observed that some choose to cooperate, but refrain from punishing non-cooperators. Hence, peer punishment generates a second-order social dilemma in which cooperators not willing to punish can second-order free ride on those who do engage in costly punishment^33^,^34^,^35^,^36^,^37^,^38^.

Social institutions provide an alternative for upholding cooperation through centralised punishment mechanisms^6^,^39^,^40^,^41^,^42^,^43^,^44^,^45^,^46^,^47^,^48^,^49^. Laws are issued to tackle tragedies of the commons like over-fishing, littering, or air pollution. Contracts are made between individuals to prevent exploitation in mutual agreements like rentals, insurances, or investments. Authorities, like courts or the police force, enforce these institutions. An essential characteristic of these institutions is that they embody a centralised power to punish^50^.

Institutions with centralized punishment can solve some of the problems related to peer punishment. For example, institutions can prevent anti-social punishment if the punishment rules in place focus on free riding, and agents cannot punish each other directly anymore. However, such institutions rely on the support of their members, and theory suggests that the second-order free rider problem is only solved if group members not willing to provide for the institution can also be punished^45^,^51^. In the experimental literature it has been demonstrated that institutions like pool punishment or the ‘hired gun’ mechanism can uphold cooperation^6^,^47^. The institutional punishment is either stronger the more was contributed to it (in the case of pool punishment), or is only executed if a certain threshold is reached (in the case of the ‘hired gun’ mechanism)^47^,^52^. In line with theoretical predictions, cooperation is particularly stable when the central institution sanctions not only free riders, but also those who refrained from supporting the institution (i.e. second-order free riders)^53^. Further, participants self-select into societies with such central institutions responsible for the punishment of free riders^54^, and they show a preference to vote for the establishment of institutions that also punish those who do not contribute to the maintenance of the institution^53^.

Centralised power, however, is also an important feature of groups that are not fully governed by laws or contracts and have not established institutions like pool punishment, ranging from hunter-gatherer chiefdoms to modern Internet communities. For example, Wikipedia provides a global public good to which everybody can contribute, but only a small share of its editors holds the right to enforce policy and sanction antisocial behaviour. Here, we focus on the process through which cooperators delegate their punishment power to a small number of punishers^55^.

We hypothesize that voluntary centralisation of punishment power can play a crucial role in sustaining cooperation in an environment where peer punishment otherwise fails, and where legal institutions are infeasible or too costly. We test this hypothesis experimentally by introducing a new experiment, which we call the ‘power transfer game’.

The power transfer game consists of the following three stages: power transfer, contribution to a public good, and costly punishment. In our experiment, participants played the game in groups of five.

In the power transfer stage, initially each group member has a power of 1 at her disposal and can give up and transfer punishment power to other group members at no direct cost. Power can be transferred in units of 0.1 and can be distributed among multiple group members. The sum of power kept and received from others determines a group member’s punishment effectiveness (defined below). After all power transfer decisions are made, everyone is informed about how much punishment power each group member has. Importantly, power transfer does not change the total punishment power in the group but (may) change its distribution among group members.

In the contribution stage, representing a standard linear public goods game, participants receive an endowment of 20 monetary units (20 MUs = 0.50€) and decide simultaneously and independently how much to contribute to a ‘group project’. Group members keep any MUs not contributed. The sum of MUs contributed to the group project is multiplied by 1.5 and distributed equally among all five group members, regardless of how much each individual contributed. This poses a social dilemma, because the return of each contributed MU is 1.5 MU for the group, but only 0.3 MU for the individual. Therefore, if all participants were selfish payoff-maximisers they should not contribute at all. In that case, each participant would earn 20 MUs. However, if all contributed their entire endowment to the group project, each participant would earn 30 MUs (20 MUs × 5 group members × 1.5 multiplier/5 group members), and group welfare would be maximised. At the end of the contribution stage, all group members are informed about how many MUs each one contributed and how many they kept for themselves.

Finally, in the punishment stage, group members are able to punish their peers. Punishment is dealt out by assigning between 0 and 10 punishment points. Punishment decisions are made simultaneously and independently. Subsequently, group members see how many punishment points each group member assigned and to whom. Punishment is costly and reduces the earnings of both the punisher and the punished. For each assigned punishment point, the punisher pays 1 MU. Here the power transfer from the first stage enters the picture as the amount of MUs that are deducted from the punished is determined by the power of the punisher. For example, if in the power transfer stage, group member A decided to transfer all of her power to group member B, and no one else transferred any power, then B would now have a power of 2, A would have a power of 0, and all other group members would still have their initial power of 1. Subsequently, if both A and B decided to punish group member C, then each point B used to punish C would lead to a reduction of 2 MUs in earnings for C, whereas each point A used to punish C would lead to no reduction in earnings for C. Both A and B would have to pay 1 MU for each punishment point that they assign to C. One way to think about power in this experiment is that it embodies a measure of social support. The more support a group member receives from other group members, the stronger is the sanctioning effect of this group member on others. Alternatively, power can be seen as a representation of the social status within the group. A game theoretic description and a detailed presentation of the computer interface can be found in the Supplementary Information.

## Experimental Implementation

In the experiment, participants (N = 350) were allocated to one of three conditions that differed in how punishment power was determined. The experiment lasted for a total of 20 rounds in fixed groups of five. In the endogenous power transfer condition (N = 135) participants repeatedly played the power transfer game described above. The remaining participants were allocated to two control conditions: the fixed condition (N = 80) or the exogenous condition (N = 135). In the fixed condition, each participant had a punishment power of 1 and participants were not able to transfer any power. Thus, each group member had a 1:1 effectiveness-to-cost ratio of punishment during the whole experiment. In the exogenous condition, power transfer was not voluntary. Instead, for each group in the endogenous condition we created a twin group in the exogenous condition, in which the history of power transfers and, hence, punishment effectiveness, was mirrored at the individual level. Thus, each group member followed the same change in punishment power across rounds as its twin.

In the endogenous treatment power transfers can make some group members more effective punishers, but group members can also select whom they want to transfer punishment power to. Therefore, with the exogenous treatment, we can test how important this voluntary selection of group members to hold punishment power is for sustaining cooperation.

**Figure 1:**
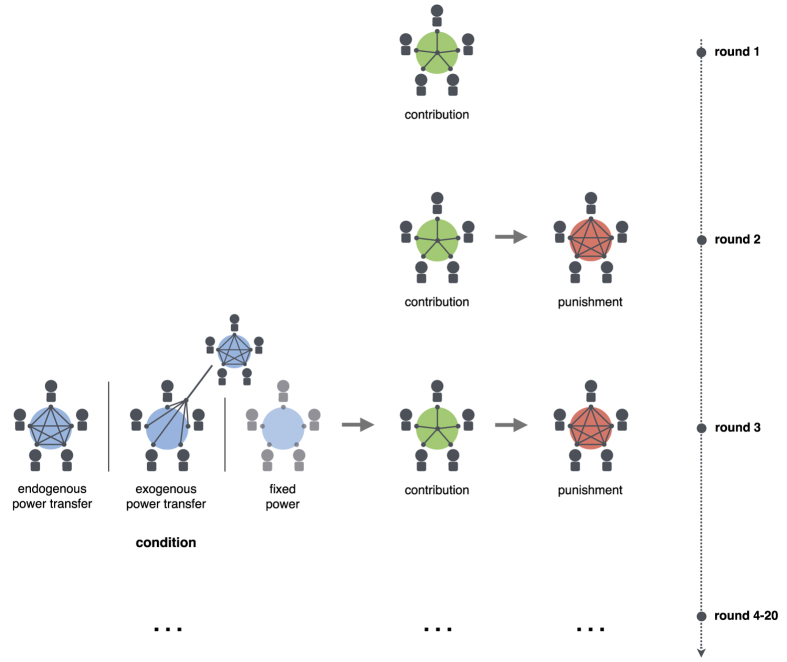
Timeline of the experiment. In all three conditions, groups started with one round of only a contribution stage, followed by round 2, consisting of a contribution stage and a punishment stage. In round 3, the experimental manipulation was introduced. In the endogenous condition, representing the power transfer game, group members were able to transfer power to other group members before the contribution and punishment stages. Each exogenous condition group mirrored the power transfers of one endogenous condition group and thus group members were not able to transfer power voluntarily. In the fixed condition, power transfers were not possible, and everyone’s power was fixed to 1. Rounds 4 to 20 had the same structure as round 3, according to the condition.

In all three conditions, the different stages of the game were introduced sequentially to the participants (Fig. 1). The experiment started with a round consisting of only a contribution stage (public goods game). The second round consisted of a contribution and a punishment stage (i.e. public goods game with punishment). In the third round, the power mechanism was introduced to the experiment according to the condition. Subsequent rounds had the same structure as the third round. Each round began with the power transfer stage. The transfer decisions made in the previous round served as the status quo for the current round. When entering a new round, participants would see the power status each group member had in the previous round together with the transfer decisions made by the participant in the previous power transfer stage. Thus, by default, the participant would make the same power allocation as she chose in the previous round. However, each participant could also decide to modify their power allocation.

## Results

**Figure 2:**
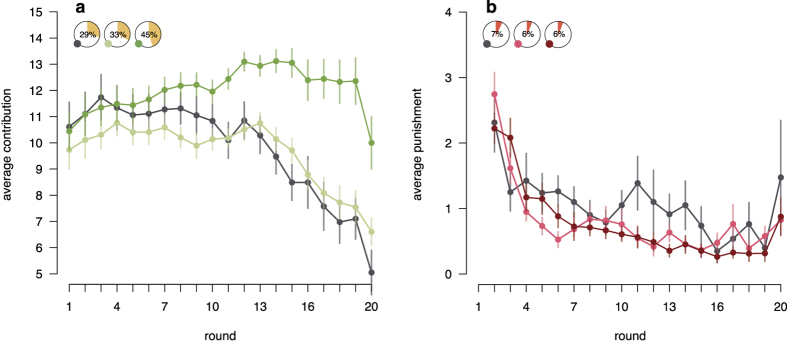
Cooperation and punishment over rounds. (**a**) Mean contributions to the group project for endogenous (dark green), exogenous (light green) and fixed (grey) conditions. Yellow pie charts show overall earnings as a percentage of the social optimum (maximum cooperation without punishment, 30 MUs per group member = 100%), compared to the selfish outcome (minimal cooperation without punishment, 20 MUs per group member = 0%) for each condition. (**b**) Average amount of MUs spent on punishment in the endogenous (dark red), exogenous (light red) and fixed (grey) conditions. Red pie charts show the average amount of MUs lost due to punishment dealt and received as a percentage of the total earnings for each condition. Error bars show the within-subject standard errors of the mean.

In all three conditions participants transferred roughly half of their endowment to the group project in the first round. In subsequent rounds, in the fixed condition with decentralised 1:1 punishment, cooperation decreased steadily (Fig. 2a; mixed effect regression, round coefficient = −0.28, 95% CI = [−0.51, −0.05], see Supplementary Information for a detailed presentation of all consecutive analyses, as well as additional supporting analyses). In contrast, in the endogenous condition with voluntary transfer of power, initial cooperation was not only sustained, but even increased slightly over time (Fig. 2a; mixed effect regression, round × endogenous condition coefficient = 0.46, 95% CI = [0.16, 0.74]). This was not the case for groups in the exogenous condition. Lacking the freedom to decide whom to transfer power to, these groups showed a decline in cooperation that was not significantly different from that in the fixed condition (Fig. 2a; mixed effect regression, round × exogenous condition coefficient = 0.22, 95% CI = [−0.06, 0.51]). Thus, only the voluntary transfer of power could sustain cooperation on a relatively high level.

The punishment histories for all three conditions are displayed in Fig. 2b. Overall, average MUs assigned for punishment declined over the course of the experiment. This decline was the strongest in the endogenous condition (mixed effect regression, round × endogenous condition coefficient = −0.07, 95% CI = [−0.13, −0.00]).

The higher levels of cooperation and the more pronounced decline in punishment led to higher group earnings in the endogenous condition. Participants in groups with the ability to transfer power earned progressively more compared to participants in the two control conditions (Fig. S13, mixed effect regression, round × endogenous condition coefficient = 1.47, 95% CI = [0.56, 2.41]; difference between round × endogenous and round × exogenous condition coefficient = 0.79, 95% CI = [0.32, 1.26]). In contrast, there was no significant difference in earnings over rounds between the fixed and exogenous condition (Fig. S13, mixed effect regression, round × exogenous condition coefficient = 0.68, 95% CI = [−0.24, 1.59]). Thus, only voluntary power transfer enabled participants to achieve earnings much closer to the social optimum.

**Figure 3:**
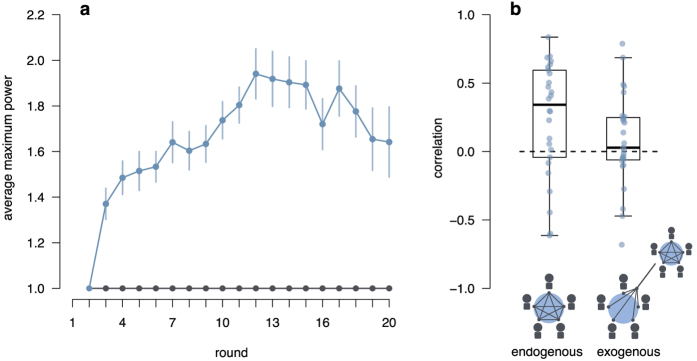
Power and cooperation. (**a**) Change of average power of the most powerful group member over rounds in the endogenous condition (blue). In the exogenous condition, power transfers were identical to the endogenous condition by construction, and thus, the average power of the most powerful group member was the same. In the fixed condition, power was fixed to 1 (grey). Error bars show the within-subject standard errors of the mean. (**b**) Distribution of correlations across rounds between maximum power and cooperation for each group in the endogenous and exogenous condition. Thick horizontal bars represent the medians.

To understand the role of voluntary power transfers in overcoming the cooperation dilemma, we looked at the pattern of power allocations that emerged over time in the endogenous treatment. A substantial fraction of participants (37%) already transferred power in the first round when power transfer was possible (round 3, see Fig. 1). Importantly, the amount of power held by the most powerful group member increased significantly over rounds (Fig. 3a, mixed effect regression, round coefficient = 0.02, 95% CI = [0.00, 0.04]), indicating that power became more centralised over the duration of the game.

The pattern of power allocations in the endogenous condition was mimicked in the exogenous condition, but only in the endogenous condition centralisation of power was positively related to cooperation. To see this, for each group we computed the correlation across rounds between power held by the most powerful group member and average cooperation. For groups who could transfer power voluntarily, higher power centralisation was associated with higher average group cooperation (Fig. 3b, mean Pearson’s r = 0.24, one-sample t-test, t(25) = 2.9, P < 0.01, two-sided). In contrast, for groups in the exogenous condition, experiencing exactly the same power centralisation but without the ability to transfer power voluntarily, correlations between power centralisation and cooperation were not significantly different from zero (Fig. 3b, mean Pearson’s r = 0.08; one-sample t-test, t(26) = 1.3, P = 0.21, two-sided).

In order to understand who transferred and who received power, how it was used, and what effect it had on group members, we analysed decisions in the endogenous condition on the individual level. Notably, although participants were unaware of the subsequent introduction of the power transfer mechanism, behaviour in the first two rounds reliably predicted a group member’s average power status later in the game. Initial cooperators, i.e. those who contributed at or above the group average in the first round, received significantly more power over the course of the experiment than initial free riders, defined as group members who contributed less than the group average (Mann-Whitney U-test, U = 2847.5, P < 0.01, two-sided). Similarly, group members who punished free riders in the first punishment stage (round 2) received significantly more power from other group members than those who did not punish (Mann-Whitney U-test, U = 2294, P = 0.02, two-sided).

Looking at power transfers from round to round shows that such transfers were mostly executed by non-punishers. Group members with a lower than average punishment expenditure in the past had a significantly higher likelihood to give up power (mixed effect logistic regression, t-1 punishment difference coefficient = 0.51, 95% CI = [0.17, 0.84]). Moreover, the likelihood of receiving power was significantly increased by being a cooperator or spending MUs on punishing free riders in the previous round (mixed effect logistic regression, t-1 cooperator coefficient = 0.52, 95% CI = [0.18, 0.86]; t-1 punishing free rider coefficient = 0.78, 95% CI = [0.39, 1.19]). In turn, gaining power further increased the odds of punishing free riders (mixed effect logistic regression, power coefficient = 1.55, 95% CI = [0.85, 2.23]) and overall expenditure on costly punishment (mixed effect regression, power change coefficient = 0.86, CI = [0.64, 1.07]). Since those willing to engage in costly punishment and cooperating above the group average were more likely to gain power, and, in turn, gaining power further increased the likelihood of spending own MUs on punishment, powerful group members earned less than the group average (correlation of power and earnings, Spearman’s rank correlation r = −0.24, P < 0.01, Fig. S17). This indicates that the behaviour of powerful group members was not driven by selfish payoff-maximization.

Group members increased their contributions in response to both punishment and power changes. In line with earlier results from experiments without power transfers, we see that the more MUs someone lost due to receiving punishment in the previous round, the more she increased her contribution to the group project (mixed effect regression, earning reduction coefficient = 0.31, 95% CI = [0.25, 0.37]). Importantly, however, we also find that the higher the increase in power centralisation from the previous round, the more group members increased their contributions compared to the previous round (mixed effect regression, power change coefficient = 4.76, 95% CI = [3.06, 6.48]). Thus, group members already reacted to the threat of powerful punishment due to power centralisation, not only to actual punishment.

Giving up power may be interpreted as delegating the responsibility to punish free riders and trying to save the cost of punishment. Such delegation and second-order free riding on those willing to punish might lead to getting sanctioned by others. We therefore tested whether transferring power increased the likelihood of getting punished in the consecutive punishment stage. However, the main predictor for getting punished was free riding on public good provisions (mixed effect logistic regression, free riding coefficient = 2.49, 95% CI = [2.18, 2.81]), while transferring power did not significantly alter the odds of getting punished (mixed effect logistic regression, power transferred coefficient = −0.44, 95% CI = [−1.02, 0.15]). Further, we tested whether initial second-order free riders-defined as those who contributed equal to or above the group average but punished below the group average in the first two rounds–were punished more over the course of the experiment compared to initial punishing cooperators, those who contributed equal or above the group average and also punished equal or above the group average in the first two rounds, and initial (first-order) free riders, those who both contributed and punished below the group average in the first two rounds. We find that this was not the case. On average, over the whole experiment, initial second-order free riders were not punished significantly more than initial punishing cooperators (Fig. S15, Dunn Test, z(2) = −0.67, P = 0.75, two-sided) and were punished less than initial first-order free riders (Fig. S15, Dunn Test, z(2) = 3.14, P <0.01, two-sided).

Not all groups in the endogenous condition were able to solve the social dilemma and it is important to understand what determines the cooperation success and failure of groups. In total, cooperation increased steadily over time in 17 out of 27 groups (cooperative groups), whereas cooperation decreased in the remaining 10 (non-cooperative groups). This increase or decrease in cooperation is not explained by initial propensities to cooperate: Cooperation in the first round was not significantly different between cooperative and non-cooperative groups (t-test, t(24) = 0.8, P = 0.43, two-sided). Hence, something else must have generated the difference in cooperation over rounds between these groups.

Several aspects may play an important role here. First, how centralised punishment power is. Second, the willingness to give up punishment power or how much power is transferred. Third, whether a suitable group member has been selected for having the most punishment power. The centralisation of punishment power was already defined above. We measure the willingness to give up power by the total amount of power transferred within the group. To evaluate whether the selection of powerful group members was successful, we calculated the share of rounds in which the group member most willing to punish free riders in the past became the most powerful.

**Figure 4:**
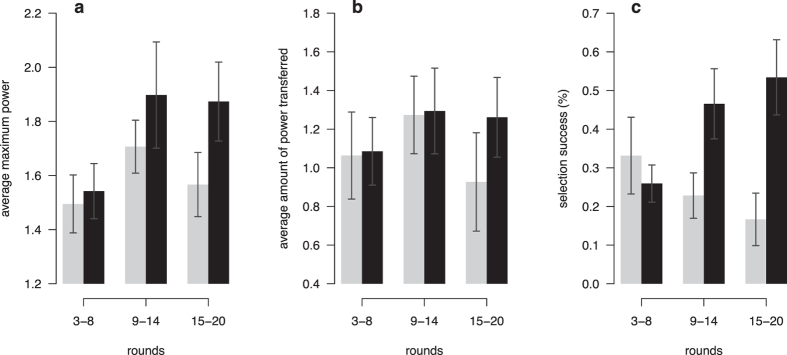
Characteristics of cooperative and non-cooperative groups across time intervals. Bars depict groups in which cooperation declined (light grey), or increased (dark grey). (**a**) Power centralisation, measured by the power of the most powerful group member; (**b**) average amount of power transferred; (**c**) selection success, measured by the share of rounds in which the most active punisher of non-cooperators of past rounds was the most powerful.

Across cooperative and non-cooperative groups, power centralisation, the willingness to give up power, as well as selection success were similar in the first third of the experiment (Fig. 4). However, power centralisation increased more sharply in cooperative groups and remained stable towards the end of the experiment (Fig. 4a), whereas it decreased in non-cooperative groups. This observed difference was not driven by willingness to give up power. The average amount of power transferred was similar in the first two thirds of the experiment (Fig. 4b). Instead, cooperative and non-cooperative groups strongly diverged in their success to centralise power in the hands of a group member who reliably punished free riders over past rounds (Fig. 4c). Thus, transferring sufficient power to the right group member was crucial for maintaining cooperation.

**Figure 5:**
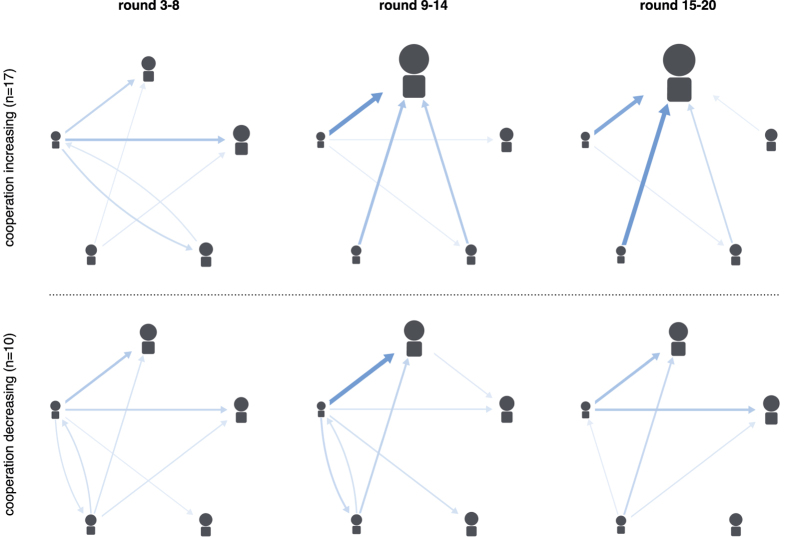
Power networks, by time interval and cooperation success. Each network shows the average power transfers (blue arrows) of groups in which either cooperation increased (top) or declined (bottom) in a given third of the experiment. The thickness of the line is proportional to the amount transferred. The size of the group members (nodes) is proportional to the amount of accumulated power.

Figure 5 shows that the power transfer networks of cooperative and non-cooperative groups were quite different. While the initial network structure was similar, non-cooperative groups diverted more power away from the centre in subsequent rounds, and also transferred it along circles, leading to less power centralisation. On the other hand, cooperative groups directed more and more power to one group member over time.

## Discussion

Voluntary centralisation of punishment power fosters cooperation and leads to a welfare increase in environments where decentralised peer punishment is unable to sustain cooperation. The transfer of power mitigates the social dilemma by enabling group members who do not punish (second-order free riders) to empower cooperators who are willing to sacrifice private resources to bring free riders in line. Free riders anticipate this behaviour and raise their cooperation when they observe that a powerful group member is emerging.

Our work demonstrates the emergence of centralised punishment out of a ‘state of nature’ characterized by weak and decentralised punishment. The resulting power hierarchy overcomes known problems of fixed peer punishment. First, the centralisation of power solves the effectiveness problem. Second, anti-social punishment can be reduced, since when pro-social punishers gain power, anti-social punishment becomes more risky. Third, those cooperating but not willing to punish, i.e. second-order free riders, can delegate their power to those willing to take over this responsibility, thereby mitigating the second-order free rider problem. While this delegation of responsibility to punish could have been perceived as an attempt to take advantage of those participants willing to engage in costly punishment, it was not sanctioned by other group members. Instead, powerful group members mainly focused their punishment on participants who were free riding on the provisions to the public good.

The results show that the most powerful group members earned the least, indicating that their behaviour was not (solely) driven by financial incentives. They were instead willing to use their power for the sake of the group by safeguarding cooperation from free riders (see Ref. 56 for a similar result in spatial interactions). This demonstrates that cooperators exist who are willing to take over the role of the punisher without a ‘salary’. Thus, with power transfers, cooperation can be sustained without a centralized punishment institution that is costly to maintain even in the absence of free riders^45^. It is essential, however, that power is concentrated in the right hands. When groups did not have the freedom to decide whom to direct power to, or failed to select the right group member, cooperation could not be sustained.

It could be argued that in most societies, high power status often confers material and social benefits to its holders. How these benefits influence group interactions in a social dilemma could be investigated with our approach in the future by, e.g., giving group members with high power a higher share of the public good or the power to also influence the allocation of the public good. Further, we did not allow participants to reject power given to them. In small self-governing groups it is often difficult to refuse the support received from others. In addition, such support might generate psychological pressures to take over responsibility and act in the interest of the group. It is an interesting open question whether group members who receive punishment power are motivated by a genuine concern for cooperation, or merely by the social expectations of their peers. By allowing group members to reject the power allocated to them, these two factors could be disentangled.

Social structures that are characterized by an unequal distribution of power are not only prevalent in human societies but also in other social animals^57^. For example, many nonhuman primates live in complex social groups organized in dominance hierarchies^58^,^59^. The emergence of social structures in which some group members have more power than others to enforce shared goals may be a crucial step in the evolution of cooperation^60^. In human societies, institutions such as elected representative bodies, legal courts and law enforcement agencies govern much of social life. These institutions are built upon and embody the centralisation of power. The willingness to give up, transfer and centralise power, demonstrated here, can be seen as an important intermediary step and prerequisite to the constitution of such complex institutions.

## Methods

Participants were recruited from the subject pool of the Behavioural and Experimental Economics Lab (BEElab) at Maastricht University and were invited via e-mail. Experiments were conducted with the informed consent of 350 healthy adult subjects who were free to withdraw from participation at any time. Only individuals who voluntarily entered the experiment recruiting database were invited, and informed consent was obtained from all participants by electronic acceptance of an invitation to attend an experimental session. The experiments were conducted following the peer-approved procedures established by Maastricht University’s Behavioral and Experimental Economics Laboratory (BEElab). Our study was approved by the BEElab at a public ethics review and project proposal meeting that is mandatory for all scholars wishing to use the BEElab facilities. A total of 350 undergraduate students (mean age = 21.1 +/− 2.6, 204 female) were randomly assigned to groups of five and allocated to one of the three different conditions that differed in how punishment power was determined. Both the endogenous and exogenous conditions were assigned 27 groups (135 participants); 16 groups (80 participants) were allocated to the fixed condition. By having a unique symbol assigned to each group member, participants could track the behaviour of other group members throughout the experiment. Each experimental session comprised at least 3 and at most 5 groups. Participants were seated in separate cubicles, where they were provided with a notepad and a pen to make notes. Sessions typically lasted for 90 minutes. Participants were paid 21€ on average.

The experiment consisted of 20 rounds. Whenever a new stage was introduced, i.e. at the beginning of rounds 1–3, participants received instructions on the computer screen and had to answer a set of comprehension questions. Instructions used neutral labels for describing the cooperation dilemma, the punishment and the power mechanism (see the Supplementary Information for details). The experiment started with one round of a public goods game, followed by one round of a public goods game with punishment. In round 3, the power transfer mechanism was introduced according to the condition. Voluntary transfer of power was only possible in the endogenous condition. In this condition, the power transfer decisions made in the previous round served as the default option for the current round. However, participants could freely change their allocation each round.

## Additional Information

**How to cite this article**: Gross, J. *et al.* Building the Leviathan – Voluntary centralisation of punishment power sustains cooperation in humans. *Sci. Rep.* **6**, 20767; doi: 10.1038/srep20767 (2016).

## Supplementary Material

Supplementary Information
