# Supplementary material for: Building the Leviathan – Voluntary centralisation of punishment power sustains cooperation in humans
Source: Sci Rep. 2016 Feb 18;6:20767. doi: 10.1038/srep20767 (PMC4757890; doi:10.1038/srep20767)
Supplement: Supplementary Information [file srep20767-s1.pdf]

Supplementary Information

Building the Leviathan – Voluntary centralisation of punishment power sustains cooperation in humans

Jörg Gross, Zsombor Z. Méder, Sanae Okamoto-Barth, Arno Riedl

Supplementary methods **1**
Game description 1
Participants 4
Experimental setup 4

Supplementary results **13**
Measurements 13
Statistical models 15

1 Supplementary methods

Game description

We introduce a new game, the 'power transfer game'. In the power transfer game, a punishment stage and a power transfer stage are added to the standard public goods game. The game-theoretic description given below first presents the standard public goods game, and then adds the punishment and the power transfer mechanisms sequentially. For equilibrium analysis, we focus on subgame perfect and trembling hand perfect equilibria.

Public goods game

The public goods game is played by $n > 2$ players. Each player has an initial endowment of E monetary units (MUs). Players decide on how much of the endowment to contribute to a public good, keeping the remaining amount. Contributions to the public good are multiplied by m, and then distributed equally among all players. We assume $m > 1$ and $\frac{m}{n} < 1$; $\frac{m}{n}$ is called the 'marginal per capita return'. Decisions on contribution are made simultaneously. The contribution of player i is denoted by c_i, with $0 \leq c_i \leq E$. After the contribution decisions are made, each player is informed about the contributions of all players, and the level of the public good.

In the public goods game, player i's payoff is given by:

$$\pi_i = E - c_i + \frac{1}{n} m \sum_{j=1}^{n} c_j.$$

Selfish payoff-maximisation prescribes a contribution of $c_i = 0$ for each individual i, as $\frac{m}{n} < 1$, and thus the private return on each invested MU is negative: $\frac{m}{n} - 1 < 0$. However, since $m > 1$, the social return of contributions is positive: $m - 1 > 0$. Therefore, social optimum, defined as the sum of all monetary payoffs, is reached when everybody contributes their full endowment, i.e. at $c_i = E$. With these contributions, the earnings of everyone will be mE. However, at individually rational contributions of zero, each player earns just their endowment E. Thus, everyone contributing their full endowment constitutes a Pareto-improvement over zero contributions. As selfish motives clash with group interests, the public goods game is a classical example of a social dilemma.

Because contributing zero is a strictly dominant strategy for every player, $c_i = 0$ for all players constitutes the only Nash-equilibrium of the game. When the public goods game is played for a finite number of periods, through a backward induction argument we get that contributions are zero for every period in the only subgame perfect equilibrium of the game.

In our experiment, the game was played with $n = 5$ players, and the contribution multiplier was set to $m = 1.5$. The initial endowment was $E = 20$, and only integer contributions were allowed.

Public goods game with punishment

A punishment option is added to the simple public goods game. Following contributions and after being informed about their peers' contributions, players simultaneously make a punishment decision. Player i can assign $0 \leq d_{ij} \leq d_{\max}$ deduction points (DP) to each other player $j \neq i$. For each DP that player i assigns to player j, player i's own payoff is reduced by the cost of punishment, pc, while the punished player j's payoff is reduced by the effectiveness of punishment pe. After punishment decisions are made, players are informed about who punished whom, and by how much (i.e. all DP assignments d_{ij}).

In the public goods game with punishment, player i's payoff is given by:

$$\pi_i = E - c_i + \frac{1}{n} m \sum_{j=1}^{n} c_j - pc \sum_{\substack{j=1 \\ j \neq i}}^{n} d_{ij} - pe \sum_{\substack{j=1 \\ j \neq i}}^{n} d_{ji}.$$

In the only subgame perfect equilibrium of the one-shot public goods game with punishment, punishments and contributions are zero. The reasoning is as follows: Once the contribution decisions are made, no selfish payoff-maximiser has any incentive to punish, because doing so would only reduce her payoffs. Therefore, it can be known from the start that $d_{ij} = 0$. Therefore, later punishment is not credible, and cannot raise contributions above $c_i = 0$. In the finitely repeated public goods game with punishment, the same backwards induction argument can be used, starting with the last period, to show that in a subgame perfect equilibrium, punishment and contribution is zero in all periods.

Almost ubiquitously in the literature, the cost of punishment is set at $pc = 1$. In the majority of experiments, the effectiveness of punishment is higher than its cost, with the most commonly used value of $pe = 3$. In our experiment, we set the *initial* effectiveness to the same level as its cost at $pe = pc = 1$ (see the next subsection). We limited the number of deduction points that could be assigned to $d_{\max} = 10$. We imposed no lower constraint on the earnings of players. Thus, players could punish and be punished below zero income.

Power transfer game

We modify the public goods game with punishment by adding an additional decision *before* the contribution decision where players may transfer their power. Power determines the effectiveness

of punishment. Players control an equal amount of power $\widehat{pe}$, and can decide to transfer it to other players. Thus, player i decides on how much power pt_{ij} to transfer to each player j (allowing for $j = i$), with $pt_{ij} \geq 0$ and $\sum_{j=1}^{n} pt_{ij} = \widehat{pe}$. Power transfer is free. Moreover, transferring power has no impact on the cost of punishment pc. The power pe_i of player i will be the total power she keeps for herself and receives from others: $pe_i = \sum_{j=1}^{n} pt_{ji}$. Players are informed about every player's power before they decide on their subsequent contributions.

In the power transfer game, player i's payoff is given by:

$$\pi_i = E - c_i + \frac{1}{n} m \sum_{j=1}^{n} c_j - pc \sum_{\substack{j=1 \\ j \neq i}}^{n} d_{ij} - pe_j \sum_{\substack{j=1 \\ j \neq i}}^{n} d_{ji}$$

To recapitulate, the items of the sum are, in turn, the initial endowment, the contribution to the public good, the individual return from the public good, the cost of punishment dealt to others, and the punishment received from others.

The cost of punishment pc is not affected by power transfers. Thus, the same argument showing that both punishments and contributions are zero in the only equilibrium of the public goods game with punishment can be extended to the power transfer game straightforwardly. Therefore, $c_i = 0$ and $d_{ij} = 0$ in the subgame perfect equilibria of the finitely repeated power transfer game. However, we get a multiplicity of equilibria, because any level of power transfers is compatible with these choices. We can get a unique equilibrium by focusing instead on trembling hand perfection. In this case, 'slips of hand' – strategies that assign a positive probability to every pure strategy – should be taken into account. Thus, a player needs to consider the possibility that she might get punished by another by mistake. If the player has transferred any power to whomever punishes her, her payoff will be lower than if she had chosen not to transfer power. Because of this potentially harmful effect of power transfers, players should not transfer power to any other player. Therefore, in the only trembling hand perfect equilibrium, we get $pe_{ij} = 0$ for each player i and other player $j \neq i$.

In our experiment, each player controlled a total power of $\widehat{pe} = 1$, so the constraint on power transfers was $\sum_{j=1}^{n} pt_{ij} = 1$.

Timeline and parameter setup

We assign each simultaneously made decision, together with the feedback/information provided after the decision to a *stage*. The timeline of the power transfer game is thus the following.

1. Power transfer stage: power transfer decision;
   players are informed about every player's effectiveness.

2. Contribution stage: contribution decision;
   players are informed about every player's contribution.

3. Punishment stage: DP assignment;
   players are informed about all DP assignments.

To summarise, our choice of parameters for the experiment was as follows:

- $n = 5$ players;
- contribution multiplier $m = 1.5$;
- initial endowment $E = 20$ MUs;
- maximum number of deduction points $d_{\max} = 10$;

- punishment cost $pc = 1$;
- own power $\widehat{pe} = 1$.

Participants

Participants were recruited from the subject pool of the behavioural and experimental economics lab (BEElab) at Maastricht University and were invited via e-mail. Participants were randomly assigned to groups of five. Each experimental session comprised at least 3 and at most 5 groups.

Participants were paid the sum of monetary units (MUs) earned over the 20 rounds (10 MUs = €0.25) plus a show-up fee of €3 and a small sum based on an incentivised social value orientation questionnaire. In three groups we had to abort the experiment because of computer malfunction. In these groups data are available for at least 14 rounds and used as such in the analysis.

Experimental setup

Participants were seated in separate cubicles and stayed there for the whole experiment. Each participant had a notepad and a pen to make notes.

The experiment started with one round of a PG. Participants read instructions explaining the rules of the PG on the computer screen. Instructions used neutral labels for describing the social dilemma: Participants were told that they would receive 20 MUs each round and that they have to decide how many MUs to contribute to a 'project'. The output of the project would then be distributed equally among all group members, irrespective of how much each member contributed. After reading the instructions, participants had to answer a set of comprehension questions about the rules of the PG.

First round: contribution

At the beginning of the first round each participant was asked to indicate how much of the 20 MUs to contribute to the project. When entering a number, the participant saw a graphical representation of the share of MUs she would contribute. After each participant in the group made a contribution decision, they saw how much each group member contributed. Group members were associated with a unique symbol by which they could be identified throughout the whole experiment. Participants saw a summary of the earnings of each group member and the outcome of the group project for this round. This summary was provided at the end of every round.

Second round: punishment

In the second round, punishment was introduced. Participants received instructions on the computer screen and answered a set of comprehension questions about the punishment rules. Instead of 'punishment' we used the neutral label 'deduction' and 'deduction points (DP)' in the instructions and throughout the experiment. Upon answering all questions correctly, participants entered the contribution stage of round 2. After getting informed about the contribution of each group member, participants simultaneously assigned between 0 and 10 deduction points to each other group member. When entering a number, participants saw the DP costs as well as the effect the punishment would have on the punished.

The deduction stage outcome summary showed which participants were punished and by whom using a graphical matrix representation. By going through the matrix by columns, participants could see how much a group member contributed in the contribution stage, how

many DPs in total this group member spent on punishing others, and how many DPs were assigned to her by the others as well as the effect it had on her earnings. By going through the matrix row-wise, participants could see how much the corresponding group member punished other group members. On this screen, participants also had the possibility to look at behaviour of previous rounds. Thus, it was possible to review past contribution and punishment decisions of each group member. The round ended with the outcome screen.

In the second round, for each assigned DP, the punisher had to pay 1 MU and the punished would lose 1 MU. With the power transfer mechanism introduced in the third round and explained below, this effectiveness-to-cost ratio of punishment could change from round to round.

Third round: power transfer

Before entering the next round, participants received the third and final set of instructions. In the fixed condition, participants were told that contribution stage and punishment stage would now be repeated for another 18 rounds. In the endogenous condition, the power transfer stage was explained to the participants. Neutral labels for power was used. Instead of 'power', we used 'deduction effectiveness' and the stage was called 'shifting stage'. After answering a set of comprehension questions about the power transfer mechanism, participants started the third round with the power transfer stage. In this stage, participants had the possibility to transfer power to other group members. Each participant had a power of 1 and could transfer power in steps of 0.1 to other group members. Power could also be distributed among multiple group members (e.g. it was possible to transfer 0.5 to one participant, 0.2 to another participant and keep 0.3 to oneself).

After every participant made their power transfer decisions, they were shown the power each group member had for this round, based on the five transfer decisions. Note that participants could not see the individual transfer decisions of the other group member but only the outcome of these decisions, i.e. the total power of each group member for this round.

In the exogenous condition, instead of transferring power by themselves, power changed exogenously based on the power transfer decisions that participants made in one of the endogenous groups. In the instructions set, participants were told that "deduction effectiveness of you and the other group members can change" from round 3 to 20. Therefore, they could not transfer power voluntarily, but only saw the changes in power at the beginning of each round, starting from round three.

Changes in power modified the effectiveness of punishment. In the fixed condition, power was fixed to 1 and transferring power was not possible, just like in round two for all conditions. In the other two conditions power could change as explained above.

Rounds 4–20

In the exogenous and endogenous condition the power transfer, the contribution and the punishment stage were repeated for the consecutive 17 rounds, with the difference that in the exogenous condition, participants saw the change in power from round to round, without being able to influence it themselves.

Each round began with the power transfer stage. The transfer decisions made in the previous round served as the status quo for the current round. When entering round 4, participants would see the power status each group member had in the previous round together with the transfer decisions made by the participant in the previous power transfer stage. Thus, by default, the participant would make the same power allocation as she chose in the previous round. However, the participant could also decide to reverse or change the previous decision by changing the

numbers below each bar accordingly (with the only constraint that the total amount of power transferred could never exceed 1).

By introducing punishment and power transfer stage round by round to the participants, we were able to measure baseline contribution and punishment rates across conditions. There should be no significant differences in average contributions in the first and average contributions and punishment in the second round between conditions, since the actual experimental manipulation started in the third round.

Computer interface

Figures S1 to S11 show what a group member would see on the computer screen in the different stages from round 3 to the beginning of round 4 in the endogenous treatment in a hypothetical round. What is shown is meant as an example to explain the computer interface and does not represent real data.

At the beginning of round 3, our group member sees the first power transfer stage screen (Figure S1). In this example, our group member decides to transfer 0.5 of her power to group member 3 and 0.2 of her power to group member 4 (Figure S2).

After all group members made their power transfer decision, the power transfer outcome screen is shown. Due to power transfers of our and the other group members, group member 3 now holds the most power, while our group member is the least powerful in the group (Figure S3).

The next screens show the contribution stage (Figure S4). Our group member decides to contribute 15 MUs to the group project (Figure S5). After all group members made their contribution decision, the contribution outcome screen is shown (Figure S6).

After the contribution stage, the group enters the deduction stage (punishment). Figure S7 shows the input screen of the deduction stage. In the first row, the contributions of each group member in the previous contribution stage is shown. By entering numbers between 1 and 10 below each column, our group member is able to assign deduction points. This is shown in Figure S8. Our group member decides to assign 2 DPs to group member 2 and 5 DPs to group member 5.

After all group members made their punishment decisions, the deduction outcome screen is shown. In this example, both our group member and group member 3 decides to punish group member 5 by 5 DPs for free riding in this round's contribution stage (Figure S9). Since our group member only has a power of 0.3, her punishment is rather ineffective. The 5 DPs costs her 5 MUs but will only reduce the earnings of group member 5 by 1.5 MUs (0.3 effectiveness times 5 DPs). In contrast, group member 3 received power from other group members and has a total power of 2. The 5 DPs assigned to group member 5 also reduces her earnings by 5 (costs of punishment), but will reduce the earnings of group member 5 more substantially. Group member 5 would lose 10 MUs due to this punishment (2.0 effectiveness times 5 DPs). The same logic applies to the punishment of group member 2 by our group member and group member 3 (Figure S9).

On the next screen the outcome of this round is shown (Figure S10). Group members see the earnings of each group member as well as the outcome of the group project.

By pressing the button at the bottom of the screen, our group member can enter the next round, starting with the power transfer stage. Previously, she decided to transfer some of her power to group members 3 and 4. This decision serves as the default option in this round's power transfer stage (Figure S11). By pressing 'accept & proceed', she would again transfer 0.5 and 0.2 of her power to these group members, respectively.

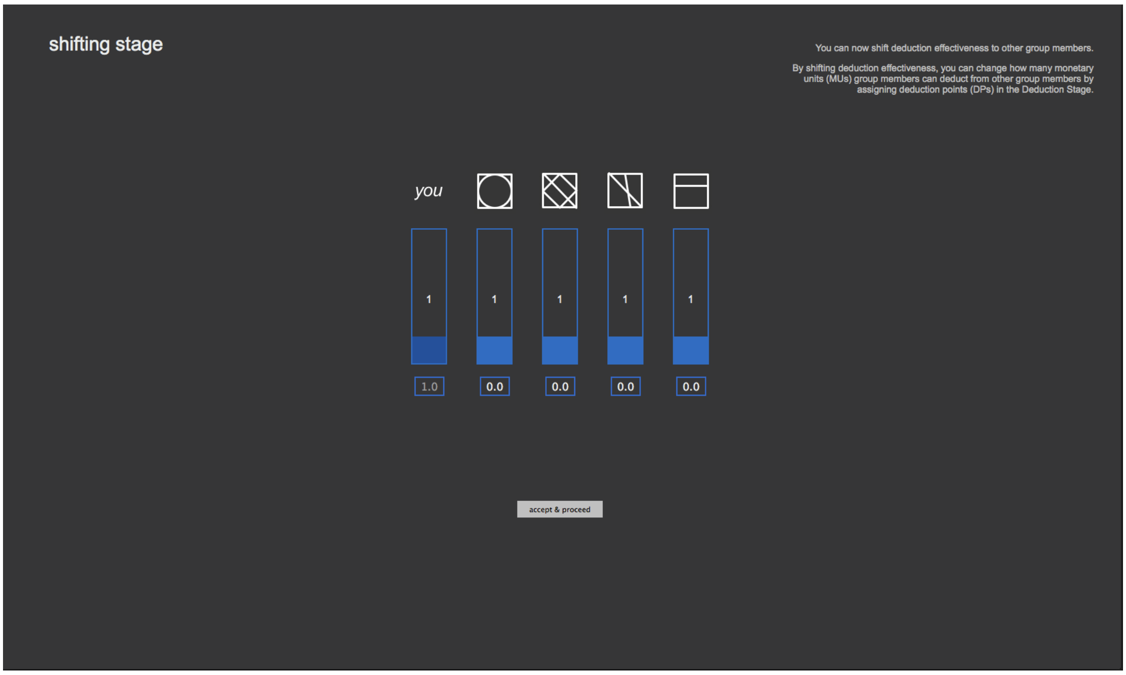

Figure S1. Power transfer stage before input, round 3. Since this is the first round with power transfers, the status quo option, visualised here, is not to transfer any power to others. Each group member is associated with a unique symbol throughout the whole experiment. Note that this screen did not appear in the fixed and exogenous condition.

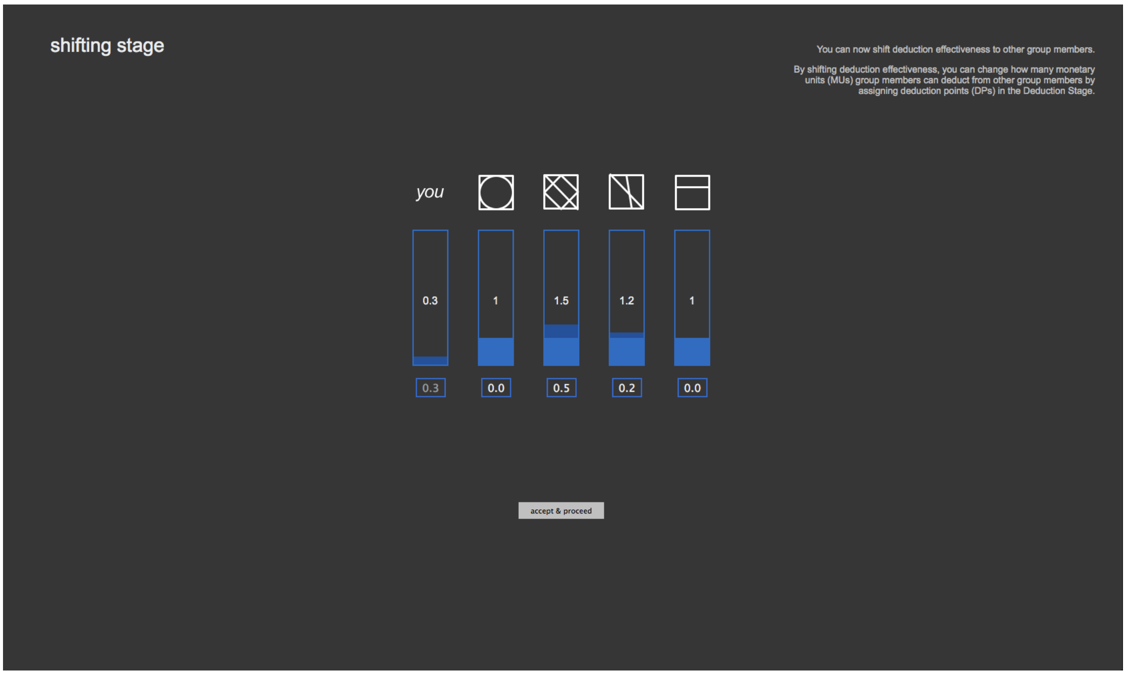

Figure S2. Power transfer stage screen, round 3. Input to each field is followed by a graphical representation of how the power status of the respective group member will change (if nobody else transfers any power). Own power is shown in dark blue. A button labelled 'accept & proceed' allows the participant to finalise her decision. Note that this screen did not appear in the fixed and exogenous condition.

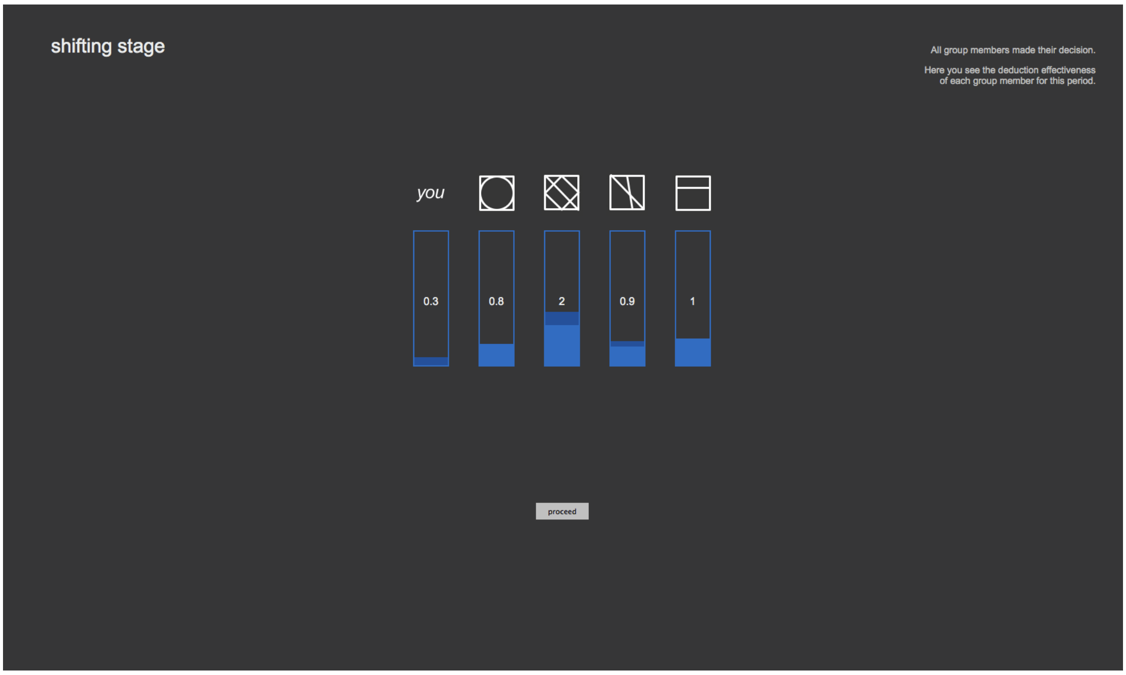

Figure S3. Power transfer outcome screen, round 3. After every group member made her power transfer decision, the power transfer outcome screen is shown. Bars represent the power of each group member for this round. Power changes due to transferring own power are shown in dark blue. Note that this screen did not appear in the fixed condition.

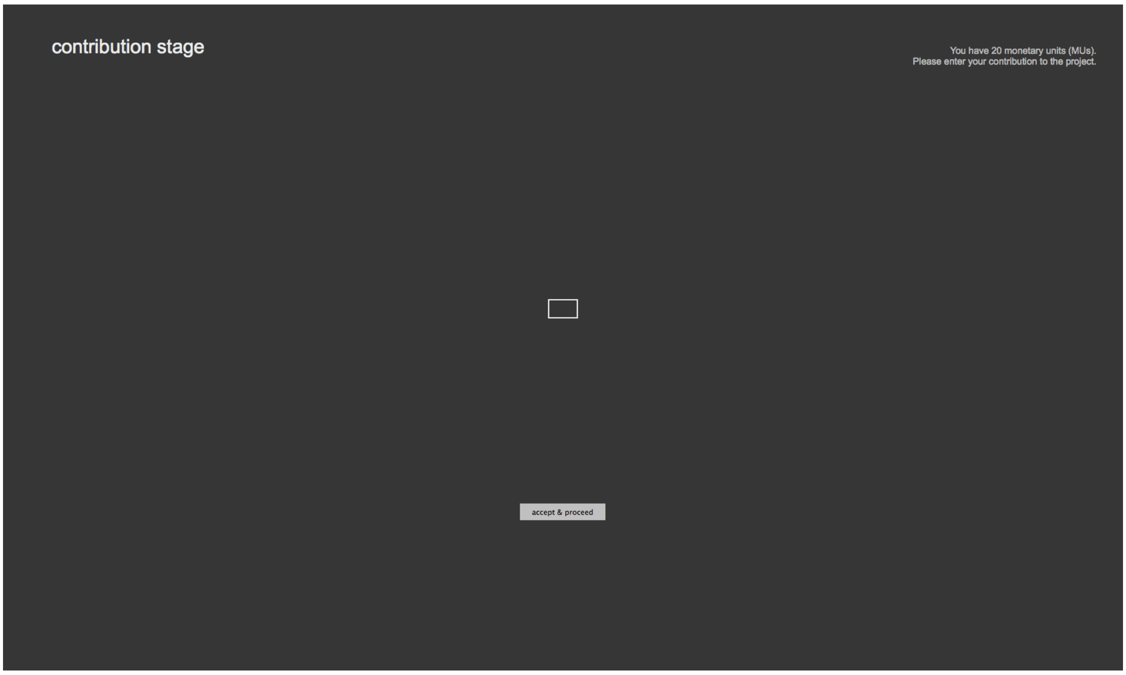

Figure S4. Contribution stage before input, round 3. Participants decide simultaneously how much of their endowment of 20 monetary units (MUs) to contribute to the group project.

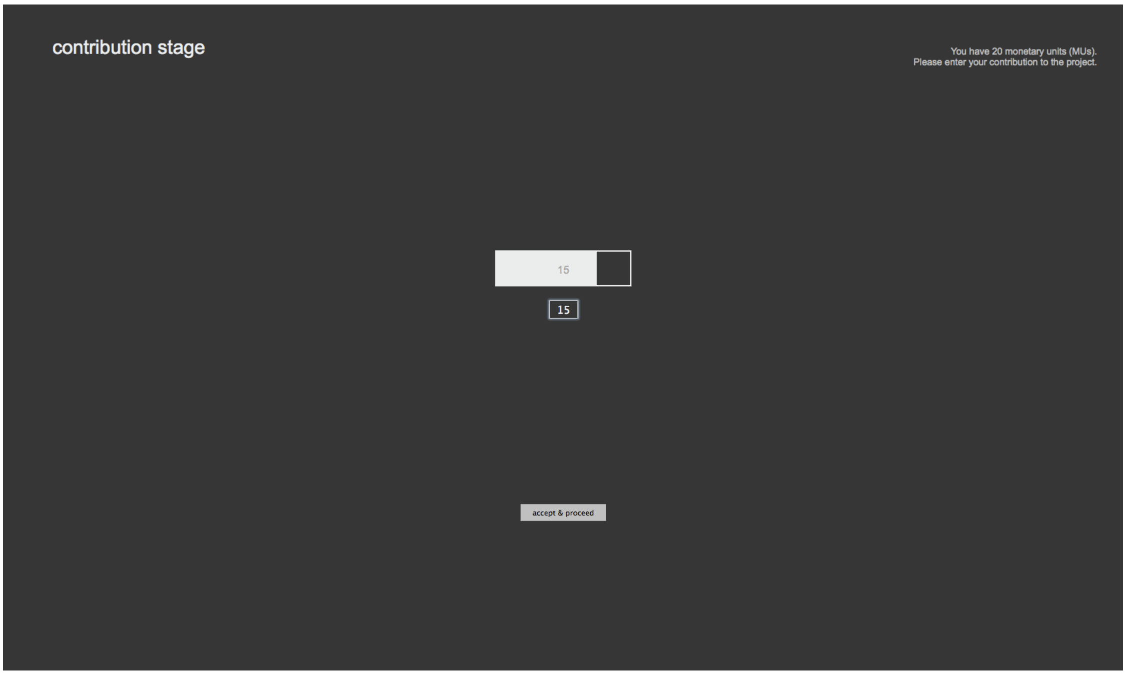

Figure S5. Contribution stage input screen, round 3. After inputting a number, the participant sees a graphical representation of the fraction of her endowment she would contribute. A button labelled 'accept & proceed' allows the participant to finalise her decision.

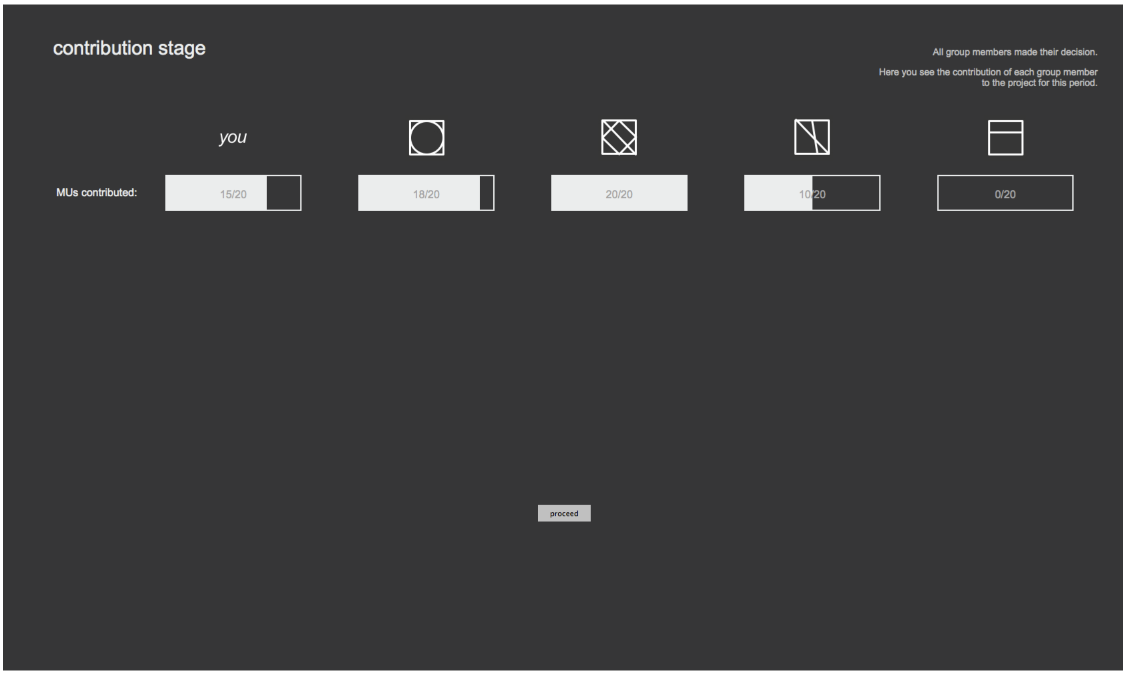

Figure S6. Contribution stage outcome screen, round 3. After every participant makes her contribution decision, the outcome screen shows how much each group member contributed.

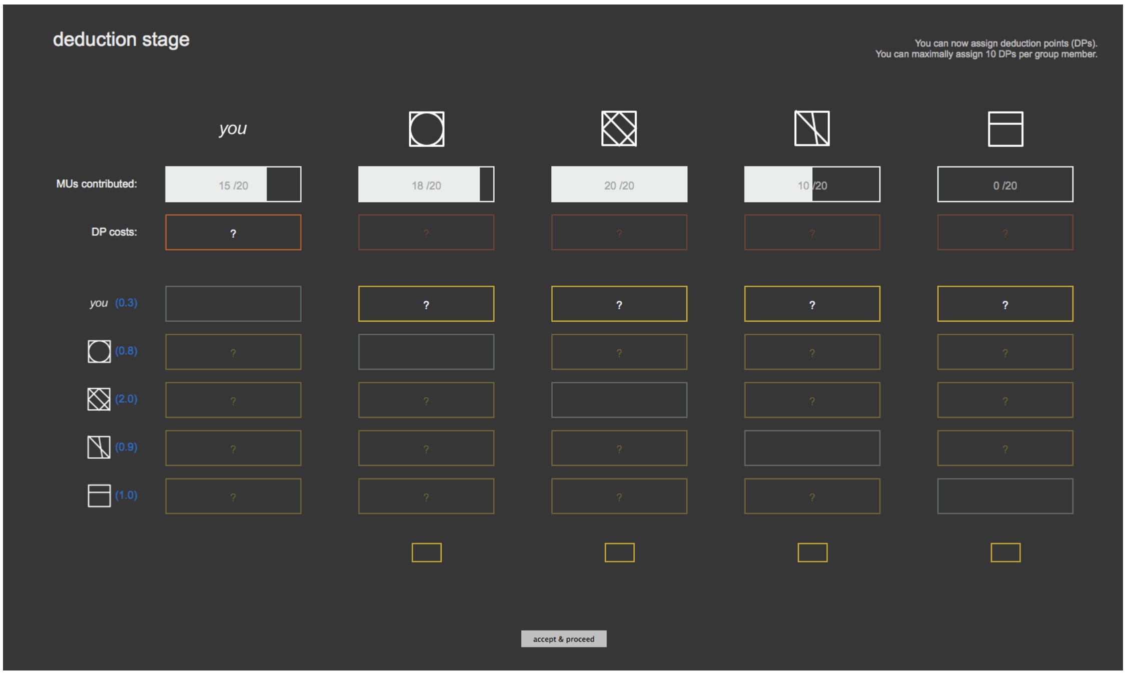

Figure S7. Punishment stage screen before input, round 3. Each participant sees a 5 × 5 punishment matrix (yellow and grey rectangles) and assigns between 0 and 10 deduction points (DPs) to each other group member. Information about the power of each group member is available (blue number next to player symbols on the left). In the first row, the contributions of each group member in the contribution stage of this round are shown.

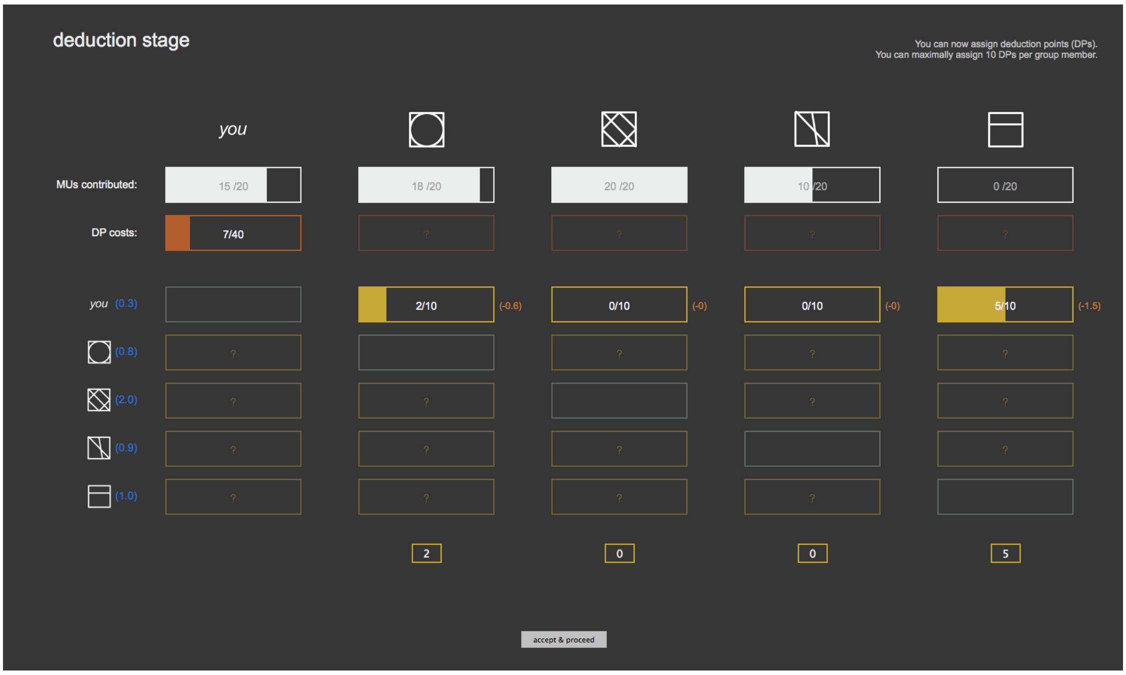

Figure S8. Punishment stage screen after input, round 3. The participant sees the total cost of her DP assignments (orange bar on the top left). The current DP assignment, as well as the effect on the group member are represented in the 5×5 matrix (yellow bars for DP assignments, orange numbers in parentheses next to them representing the effect on the punished).

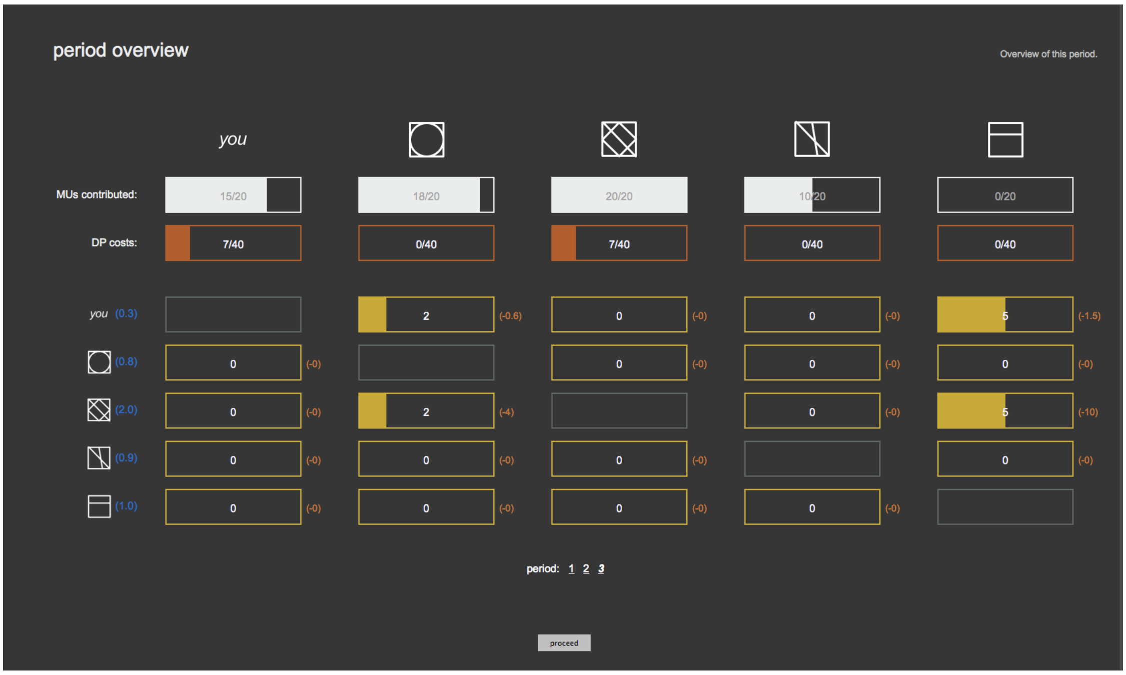

Figure S9. Punishment stage outcome screen. Participants see who was punished by whom. Each column indicates by how much a group member was punished, while the rows indicate by whom these deduction points were assigned by.

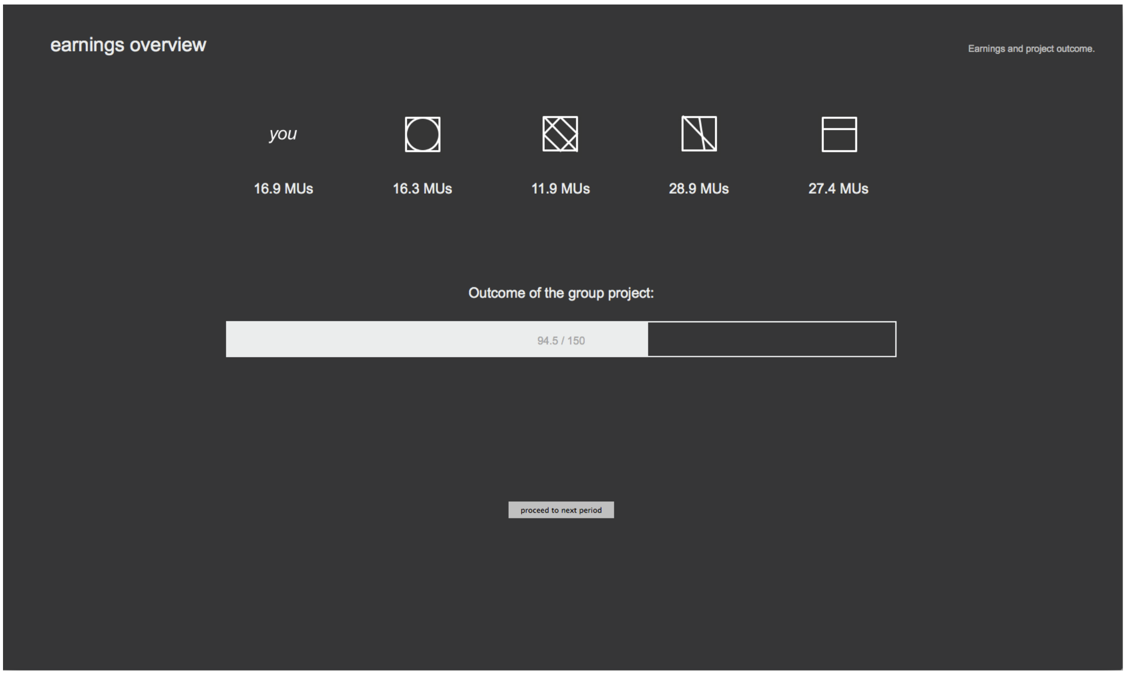

Figure S10. Earnings screen, round 3. This screen is shown at the end of each round summarising earnings and the outcome of the group project (sum of contributions ×1.5). The sum of MUs kept, MUs received from the group project, losses due to assigning DPs, and losses due to receiving punishment led to the payoffs seen on the screen.

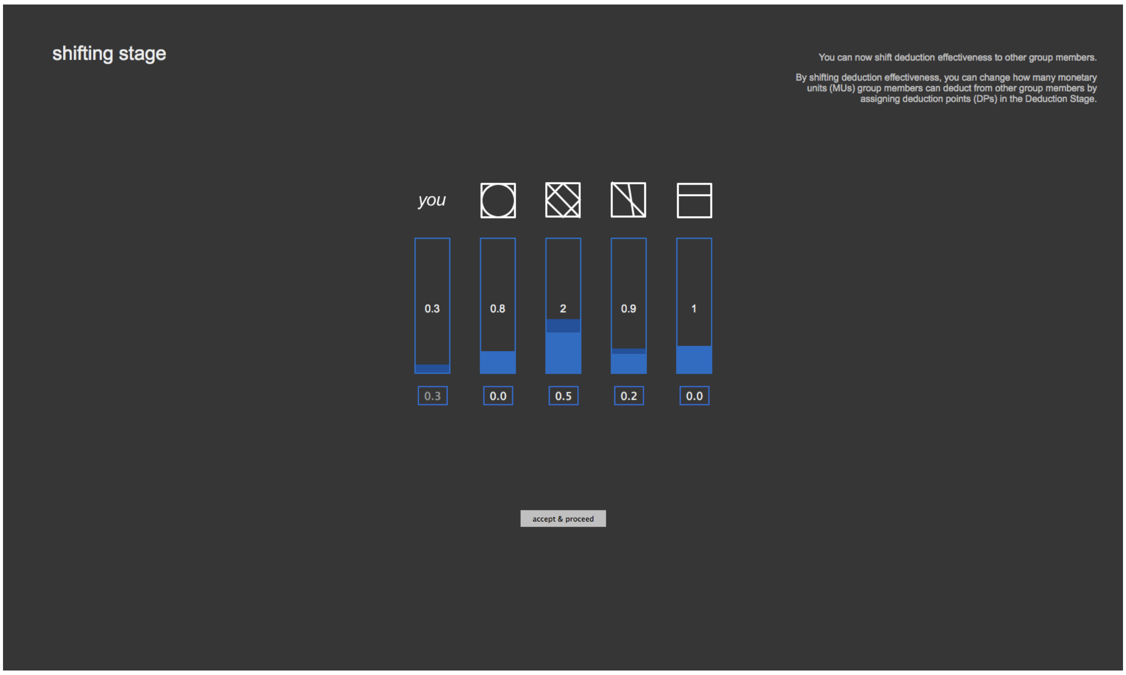

Figure S11. Power transfer stage screen before input, round 4. Each bar represents the power of each group member in the previous round. The power transfers made in the previous round serve as the status quo allocation for this round. Dark blue bar segments indicate the power allocated by this group member. Thus by pressing 'accept & proceed', the participant will again allocate 0.5 of her power to group member 3 and 0.2 of her power to group member 4.

2 Supplementary results

Measurements

For the statistical analysis, we defined proxies and compound measures based on the behavioural data. Each of these measures is defined below.

Punishment behaviour

For the receiving power and use of power models (see below), we were interested in how power was used with regards to punishment. We wanted to differentiate the punishment of free riders from the punishment of cooperators (antisocial punishment). Moreover, we opted for a measure that assigned higher value for punishing non-cooperators who deviated more from average contributions. Punishment behaviour was defined as:

$$\text{punishment behaviour}_{it} = \sum_{\substack{j=1 \\ i \neq j}}^{5} \left(\frac{\bar{c}_t - c_{jt}}{\sigma_{ct}} \times d_{ijt} \right)$$

$$\begin{aligned} \text{where } c_{jt} &= \text{contribution of player } j \text{ in round } t, \\ \bar{c}_t \text{ and } \sigma_{ct} &= \text{mean and standard deviation of contributions,} \\ d_{ijt} &= \text{deduction points assigned by } i \text{ to } j, \\ &\quad \text{in round } t, \text{ with } 2 \leq t \leq 20. \end{aligned} \tag{1}$$

Thus, for each group member in each round, assigned deduction points were weighted by the standardised cooperation of the punished group member and summed up. Negative numbers therefore indicated that punishment was predominantly used to punish cooperators (antisocial punishment), while positive numbers indicated that punishment was predominantly used to punish free riders (e.g. a punishment behaviour value of 1 can be interpreted as using one deduction point to punish a group member with a contribution that was 1 standard deviation below group average in this round's contribution stage). Figure S12 shows the percentage of punishment behaviour types across treatments. Antisocial punishment (free riders punishing cooperators) was observed less frequently than no punishment and the punishment of free riders.

Power centralisation

We summarised power centralisation through a single indicator: the power of the most powerful group member. In the fixed condition every group member had a fixed power of 1. For the endogenous and exogenous conditions, power centralisation was defined by:

$$\text{power centralisation}_t = \max_{i \in 1,\dots,5} pe_{it}$$

$$\begin{aligned} \text{where } pe_{it} &= \sum_{j=1}^{5} pt_{jit}, \text{ power (i.e. punishment effectiveness) of } i, \\ &\quad \text{in round } t, \text{ with } 3 \leq t \leq 20. \end{aligned} \tag{2}$$

A minimal power centralisation of 1 was achieved by, for instance, no power being transferred in a given round. A full power centralisation value of 5 would be achieved if all group members would transferred their power to a single individual.

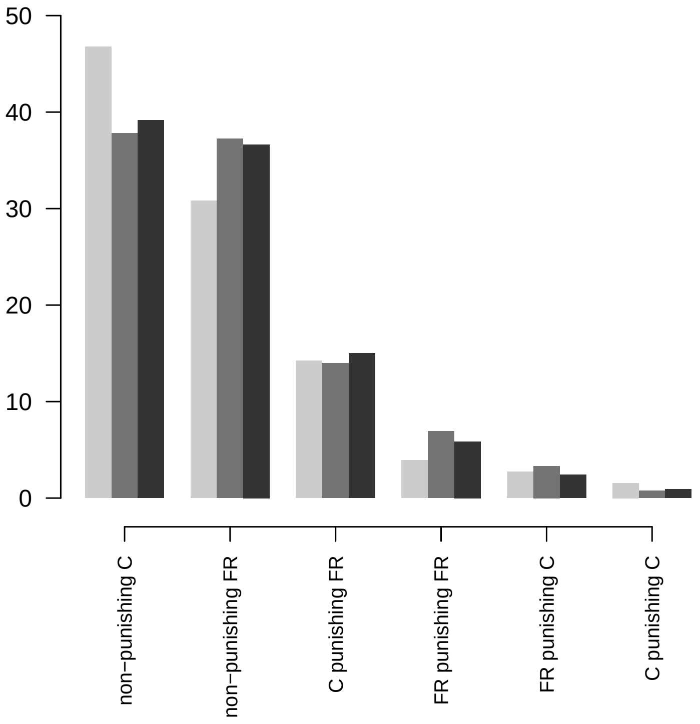

Figure S12. Cooperation and punishment decisions. Percentage of punishment decisions observed across treatments (light grey = endogenous condition, grey = exogenous condition, dark grey = fixed condition). C = cooperator, FR = free rider.

Willingness to transfer power

We defined our measure for the willingness to transfer power as the sum of all power transferred in a certain round.

$$\text{willingness to transfer}_t = \sum_{i=1}^{5} \sum_{\substack{j=1 \\ j \neq i}}^{5} pt_{jit} \qquad (3)$$
$$\text{where } pt_{jit} = \text{power transferred from } j \text{ to } i, \text{ in round } t, \text{ with } 3 \leq t \leq 20.$$

In rounds when group members did not transfer any power between them, our willingness to transfer measure took a value of 0. A willingness to transfer of 5 could be achieved by everyone transferring all their power.

Selection success

We introduced a measure to gauge a group's ability to select the most active prosocial punishers as the recipient of power transfers. First, building on Definition 1, we defined the aggregate (past) punishment behaviour as:

$$\text{aggregate punishment behaviour}_{it} = \sum_{j=2}^{t-1} \Big(\text{punishment behaviour}_{ij}\Big) \tag{4}$$
$$\text{in round } t, \text{ with } 3 \leq t \leq 20.$$

This variable summarised information about the past behaviour of group members, as potential leaders of the in-group power hierarchy. Within a group, larger values for a certain member i indicated both the amount of resource i sacrificed for punishment, as well as her propensity to pick less cooperative group members, as a target for her punishment. If the past is a reliable predictor of an individual's future behaviour, those who showed a willingness to punish non-cooperators should receive the most power. Thus, for groups in the endogenous condition, we defined our selection success in the following manner:

$$\text{selection success}_t = \begin{cases} \frac{1}{|\arg\max\limits_{i=1,\dots,5} pe_{it}|} & \text{if aggregate punishment behaviour}_{it} \in \arg\max\limits_{i=1,\dots,5} pe_{it}, \\ 0 & \text{otherwise,} \end{cases} \tag{5}$$
$$\text{in round } t, \text{ with } 3 \leq t \leq 20.$$

Specifically, if in a certain round individual i having the highest aggregate punishment behaviour was strictly more powerful than all other group members, then for that round the selection success of the group would be 1. If, however, i was less powerful than some other group member, we would assign it a value of 0. In case of a tie, the value would be 1 divided by the number of group members that are equally (most) powerful. In such a case, the group has partially solved the selection problem, and we would assign $\frac{1}{2}$, $\frac{1}{3}$, etc. to the selection success variable.

Statistical models

Because of the hierarchical structure of the data (participants clustered in groups and repeated measures over rounds), we fitted Bayesian mixed effects models to the data using R and JAGS.

Non-informative Gaussian priors (m=0, sd=100) were used for each predictor and non-informative uniform priors (range 0 to 100) for the error terms. In every model, random intercepts and slopes were allowed to covary. Therefore, the variance-covariance matrix was estimated alongside the fixed and random coefficients. For the correlations between random effects, non-informative uniform priors (range -1 to 1) were used. We used three parallel chains. For every estimated coefficient, the potential scale reduction factor (Gelman and Rubin Diagnostic) was below 1.05, indicating good mixing of the three chains and thus high convergence. Regression tables reported below show estimated coefficients together with the 95% confidence interval (CI, also called highest density interval in the Bayesian framework). Note that, since non-informative priors were used, a 95% CI that only contains negative or positive values can be interpreted as significant at a p = .05 two-sided threshold from frequentist perspective. Fitting the models using restricted maximum likelihood (REML) as implemented in the lme4 package in R revealed similar estimates and the same statistical inferences. However, models on the individual subject level failed to converge and also the censoring in the data could not be accounted for in these models.

Group level analysis

The aim of the group level analysis was to compare cooperation (i.e. contribution to the group project), punishment and earnings across the three different conditions, as well as to analyse the increase of maximum power over rounds in the endogenous condition (and thus also the exogenous condition). For this, we aggregated the data by group members, such that for each group we had one data point for each round (e.g. average contribution).

Contribution The fixed part of the contribution model contained two dummy variables coding the three experimental conditions (with the fixed condition as baseline), a continuous round predictor and the round × condition interactions. The random part contained a random intercept as well as a random slope for the round predictor for each group. Thus, for each group a separate baseline cooperation rate in round 1, and a separate slope of cooperation over rounds was estimated (see Equation 6). Since average group contribution could not exceed 20 and fall below 0, the data were treated as left and right censored.

$$
\begin{aligned}
y_i &\sim N(\mu_y, \sigma_y^2), \text{for } i = 1, \dots, n \\
\mu_y &= \alpha_{1j} + \beta_{1j}\text{round} + \alpha_2 + \beta_2\text{round} \\
&\quad + \beta_3\text{exogenous} + \beta_4\text{endogenous} \\
&\quad + \beta_5\text{exogenous} \times \text{round} + \beta_6\text{endogenous} \times \text{round} \\
\begin{pmatrix} \alpha_{1j} \\ \beta_{1j} \end{pmatrix} &\sim N\left(\begin{pmatrix} 0 \\ 0 \end{pmatrix}, \begin{pmatrix} \sigma_{\alpha_1}^2 & \rho\sigma_{\alpha_1}^2\sigma_{\beta_1}^2 \\ \rho\sigma_{\alpha_1}^2\sigma_{\beta_1}^2 & \sigma_{\beta_1}^2 \end{pmatrix} \right), \text{for } j = 1, \dots, J,
\end{aligned}
\tag{6}
$$

$$
\begin{aligned}
\text{where } n &= \text{ number of observations,} \\
J &= \text{ number of groups.}
\end{aligned}
$$

Table S1 shows the estimated coefficients together with the 95% CI. First round cooperation was predicted to be 12.6 for the fixed condition. Groups in the exogenous and endogenous condition did not significantly differ from this initial level of cooperation (β_3 and β_4). In the fixed condition, there was a significant drop in cooperation over rounds (β_2). The drop in cooperation was not significantly different in the exogenous condition (β_5). In contrast, in the endogenous condition, cooperation over time was significantly higher (β_6).

Examining the posterior distributions of the exogenous × round and endogenous × round parameter revealed an estimated difference of 0.24 with a 95% CI ranging from 0.08 to 0.39. Thus, also compared to the exogenous condition, cooperation over time was significantly higher in the endogenous condition.

Punishment The punishment model followed the same structure as the contribution model (see Equation 6), except for average deduction points spent as the dependent variable. Since average group punishment could not fall below 0, the data were treated as left censored.

Table S2 shows the estimated coefficients together with the 95% CI. Punishment expenses in the second round (first round with punishment) did not significantly differ across conditions (α_2, β_3 and β_4). In the fixed condition, the use of punishment dropped over rounds (β_2). Groups in the exogenous condition did not deviate significantly from this trend (β_5). In the endogenous condition, the drop in punishment expenses over rounds was significantly higher compared to the fixed condition (β_6).

Table S1. Contribution regression model.
Dependent variable: Contribution group average clustered by group.

		estimate	95% CI
α_2	intercept (fixed condition, round 1)	12.60	[10.19, 14.95]
β_2	round	-0.28	[-0.51, -0.05]
β_3	exogenous condition dummy	-1.84	[-4.86, 1.01]
β_4	endogenous condition dummy	-1.47	[-4.56, 1.46]
β_5	exogenous condition $\times$ round	0.22	[-0.06, 0.51]
β_6	endogenous condition $\times$ round	0.46	[0.16, 0.74]
$\sigma^2_{\alpha_1}$	error-term random intercepts	0.47	[0.38, 0.56]
$\sigma^2_{\beta_1}$	error-term round slopes	2.41	[2.31, 2.51]
σ^2_y	error-term y	4.55	[3.76, 5.42]
ρ	correlation between random effects	-0.15	[-0.40, 0.09]

Table S2. Punishment regression model.
Dependent variable: Average punishment expense clustered by group.

		estimate	95% CI
α_2	intercept (fixed condition, round 2)	1.32	[0.70, 1.94]
β_2	round	-0.08	[-0.13, -0.03]
β_3	exogenous condition dummy	-0.19	[-0.97, 0.58]
β_4	endogenous condition dummy	0.10	[-0.66, 0.89]
β_5	exogenous condition $\times$ round	-0.01	[-0.08, 0.05]
β_6	endogenous condition $\times$ round	-0.07	[-0.13, -0.002]
$\sigma^2_{\alpha_1}$	error-term random intercepts	0.08	[0.05, 0.10]
$\sigma^2_{\beta_1}$	error-term round slopes	1.47	[1.39, 1.55]
σ^2_y	error-term y	1.04	[0.78, 1.32]
ρ	correlation between random effects	-0.60	[-0.82, -0.35]

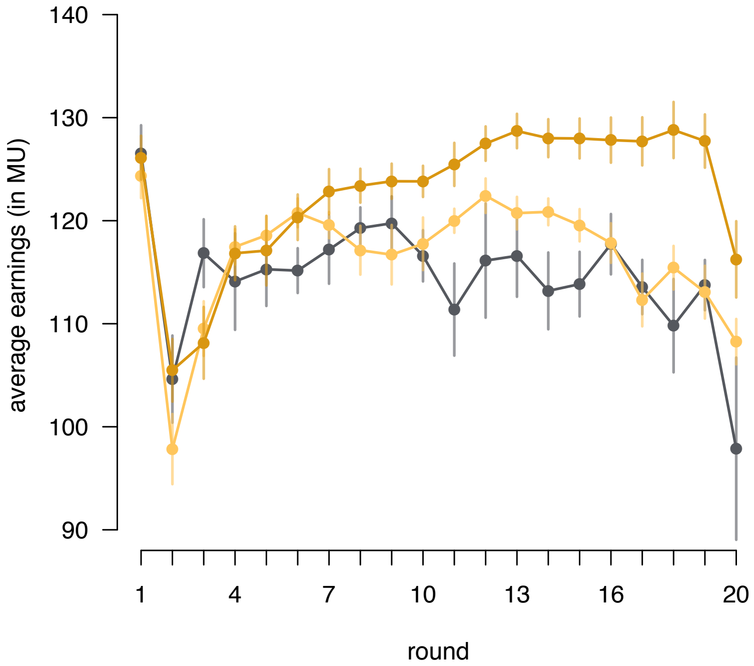

Figure S13. Average earnings over rounds. Average earnings in the fixed condition (grey), exogenous condition (light yellow) and endogenous condition (dark yellow). Error bars show the within-subject standard errors of the mean.

Group earnings Figure S13 shows the average earnings over rounds by condition. There was a substantial drop in earnings in all three conditions after the introduction punishment to the experiment in round 2, as it is often observed in PG experiments with punishment.

The group earnings regression model followed the same structure as the contribution model (see Equation 6), except for aggregated earnings (in MUs) as the dependent variable. Since earnings for one round could not exceed 150 MUs (20 MUs $\times$ 5 participants $\times$ 1.5), the data were treated as right censored.

Table S3 shows the estimated coefficients together with the 95% CI. Earnings in the first round did not significantly differ between fixed and exogenous condition, as well as fixed and endogenous condition (β_3 and β_4). Earnings were significantly higher over rounds in the endogenous condition compared to the fixed condition (β_6).

Examining the posterior distributions of the exogenous $\times$ round and endogenous $\times$ round parameter revealed an estimated difference of 0.79 with a 95% CI ranging from 0.32 to 1.26. Thus, also compared to the exogenous condition, earnings over time were significantly higher in the endogenous condition.

Maximum power The maximum power regression model followed the same random structure as the contribution model (see Equation 6), but since power transfers only happened in the endogenous condition and exogenous condition groups merely mimicked these transfers over time, the model used only data of the endogenous condition and therefore contained only one predictor coding the round. The dependent variable was power centralisation, i.e. the amount of power of the most powerful group member in a particular round. Since maximum power could not fall below 1, the data were treated as left censored.

Table S4 shows the estimated coefficients together with the 95% CI. Already in the first power transfer stage (round 3), average power centralisation was predicted to be 1.5 by the

Table S3. Earnings regression model.
Dependent variable: Average earnings clustered by group.

		estimate	95% CI
α_2	intercept (fixed condition, round 1)	118.72	[111.12, 127.07]
β_2	round	-0.36	[-1.08, 0.36]
β_3	exogenous condition dummy	-3.31	[-13.29, 6.79]
β_4	endogenous condition dummy	-3.30	[-13.30, 6.64]
β_5	exogenous condition × round	0.68	[-0.24, 1.59]
β_6	endogenous condition × round	1.47	[0.56, 2.41]
$\sigma^2_{\alpha_1}$	error-term random intercepts	1.36	[1.06, 1.68]
$\sigma^2_{\beta_1}$	error-term round slopes	13.58	[13.01, 14.15]
σ^2_y	error-term y	14.91	[12.01, 18.04]
ρ	correlation between random effects	-0.24	[-0.50, 0.02]

Table S4. Power change regression model.
Dependent variable: maximum power clustered by group.

		estimate	95% CI
α_2	intercept (round 3)	1.455	[1.237, 1.672]
β_2	round	0.024	[0.004, 0.043]
$\sigma^2_{\alpha_1}$	error-term random intercepts	0.041	[0.023, 0.060]
$\sigma^2_{\beta_1}$	error-term round slopes	0.579	[0.536, 0.624]
σ^2_y	error-term y	0.499	[0.314, 0.699]
ρ	correlation between random effects	-0.063	[-0.574, 0.465]

model (α_2). Power accumulation increased over rounds. In each round, the maximum power was estimated to increase by 0.02 on average (β_2).

Individual level analysis

The aim of the the individual level analysis was to analyse who received power, who was willing to give up power and how power affected contributions in the endogenous condition on a round-by-round basis. Therefore, the data were not aggregated on a group level and thus was clustered by groups and by individuals (over time). The regression models accounted for that by having two grouping levels (see below).

In the receiving, giving, and use of power models, the distributions of the dependent variables were non-normal and highly restricted. We therefore transformed the dependent variable in these models into a dichotomous variable and fitted logistic regressions.

Also some predictors were transformed to dichotomous 'type' variables. This has the downside of losing some statistical power, as well as the ability to make more detailed quantitative statements (e.g. with a one point increase in the independent variable, the probability of the

dependent variable being 1 changes by x). On the other hand, coefficients can be interpreted more easily. For example, by converting contributions to a binary variable (at or above/below group average), participants are classified into free riders (those who contributed less than group average) and cooperators (those who contributed at least or above group average).

Receiving power The dependent variable of the receiving power model was power received by other group members (0 = no power received, 1 = power received). The fixed part of the model contained the continuous round predictor, a dummy predictor indicating free riding or cooperation (0 = below average contribution, 1 = equal or above average contribution) in previous round's contribution stage, and a dummy predictor coding the punishment behaviour (see Equation 1; 0 antisocial or no punishment, 1 = punishment of free riders) of previous round's punishment stage as predictors.

The random part of the model contained a random intercept and a random slope for the round predictor for each group, as well as a random intercept and a random slope for the round predictor for each participant. Thus, for each group a separate baseline of the likelihood of power transferring in round 3 (first power transfer stage), and a separate slope in how the likelihood of transferring power changed over rounds was estimated. Separately, the model estimated the likelihood of power being transferred in round 3, as well as the change in the likelihood of receiving power over rounds for each participant (see Equation 7).

$$
\begin{aligned}
Pr(y_i = 1) &\sim \text{logit}^{-1}(\mu_y), \text{for } i = 1, \ldots, n \\
\mu_y &= \alpha_{1j} + \beta_{1j}\text{round} + \alpha_{2k} + \beta_{2k}\text{round} \\
&\quad + \alpha_3 + \beta_3\text{round} + \beta_4\text{contribution type}_{t-1} \\
&\quad + \beta_5\text{punishment type}_{t-1} \\
\begin{pmatrix} \alpha_{1j} \\ \beta_{1j} \end{pmatrix} &\sim N\left(\begin{pmatrix} 0 \\ 0 \end{pmatrix}, \begin{pmatrix} \sigma^2_{\alpha_1} & \rho_1\sigma^2_{\alpha_1}\sigma^2_{\beta_1} \\ \rho_1\sigma^2_{\alpha_1}\sigma^2_{\beta_1} & \sigma^2_{\beta_1} \end{pmatrix} \right), \text{for } j = 1, \ldots, J, \\
\begin{pmatrix} \alpha_{2k} \\ \beta_{2k} \end{pmatrix} &\sim N\left(\begin{pmatrix} 0 \\ 0 \end{pmatrix}, \begin{pmatrix} \sigma^2_{\alpha_2} & \rho_2\sigma^2_{\alpha_2}\sigma^2_{\beta_2} \\ \rho_2\sigma^2_{\alpha_2}\sigma^2_{\beta_2} & \sigma^2_{\beta_2} \end{pmatrix} \right), \text{for } k = 1, \ldots, K, \\
\text{where } n &= \text{ number of observations,} \\
J &= \text{ number of groups,} \\
K &= \text{ number of subjects.}
\end{aligned}
\tag{7}
$$

Table S5 shows the estimated coefficients together with the 95% CI as well as the odds ratio (exponential of coefficient). According to the model, cooperators had a 68% increase in odds of receiving power (β_4). The odds of receiving power more than doubled for participants who punished free riders in the previous punishment stage (β_5).

Giving away power The regression model for giving away power followed the same random structure described in Equation 7. The dependent variable coded the transfer of power to other group member (0 indicated no transfer of power and 1 indicated transfer of power).

In this analysis, we were interested in whether the willingness to spend points on punishment predicted the likelihood of giving away power. Therefore, we used the difference in the amount of points spent on punishment compared to group average as a dummy predictor (0 = punishment expense equal or above group average, 1 = punishment expense below group average). The

Table S5. Receiving power regression model.
Dependent variable: power received (0 = no, 1 = yes) clustered by group and participant.

		estimate	odds ratio	95% CI
α_3	intercept (round 3)	0.16		[-1.13 1.53]
β_3	round	-0.14	0.87	[-0.25 -0.03]
β_4	contribution type$_{t-1}$	0.52	1.68	[0.18 0.86]
β_5	punishment type$_{t-1}$	0.78	2.18	[0.39 1.19]
$\sigma^2_{\alpha_1}$	error-term random intercepts (group level)	3.23		[2.08, 4.53]
$\sigma^2_{\beta_1}$	error-term round slopes (group level)	2.54		[1.87, 3.26]
$\sigma^2_{\alpha_2}$	error-term random intercepts (individual level)	0.23		[0.13, 0.33]
$\sigma^2_{\beta_2}$	error-term round slopes (individual level)	0.27		[0.19, 0.34]
σ^2_y	error-term y	49.97		[1.39, 96.31]
ρ_1	correlation random effects (group level)	-0.55		[-0.89, -0.14]
ρ_2	correlation random effects (individual level)	-0.46		[-0.72, -0.18]

fixed part of the model also contained the continuous round predictor and a dummy predictor indicating free riding or cooperation (0 = below average contribution, 1 = equal or above average contribution) in the previous round's contribution stage.

Table S6 shows the estimated coefficients together with the 95% CI as well as the odds ratio. According to the model, punishing below group average increased the odds of transferring power to other group members in the next round by 67% (β_5).

Table S6. Giving away power regression model.
Dependent variable: Power giving (0 = no power was transferred, 1 = power was transferred) clustered by group and participant.

		estimate	odds ratio	95% CI
α_3	intercept (round 3)	-1.42		[-2.52, -0.41]
β_3	round	-0.04	0.96	[-0.13, 0.04]
β_4	contribution difference$_{t-1}$	0.03	1.03	[-0.32, 0.38]
β_5	punishment difference$_{t-1}$	0.51	1.67	[0.17, 0.84]
$\sigma^2_{\alpha_1}$	error-term random intercepts (group level)	1.08		[0.04, 2.17]
$\sigma^2_{\beta_1}$	error-term round slopes (group level)	4.55		[3.48, 5.67]
$\sigma^2_{\alpha_2}$	error-term random intercepts (individual level)	0.07		[0.00, 0.16]
$\sigma^2_{\beta_2}$	error-term round slopes (individual level)	0.34		[0.26, 0.42]
σ^2_y	error-term y	49.96		[0.39, 95.35]
ρ_1	correlation random effects (group level)	-0.19		[-1.00, 0.83]
ρ_2	correlation random effects (individual level)	-0.64		[-0.83, -0.44]

Use of power The regression model for use of power followed the same random structure described in Equation 7. The dependent variable coded the punishment behaviour (0 indicated no punishment or antisocial punishment and 1 indicated punishment of free riders).

As predictors we used the continuous round predictor, the group contribution of the present round and a dummy variable indicating whether the participant had power above 1 in the present round (0 = power below or equal 1, 1 = power more than 1), as well as the interaction of this dummy with the group contribution.

Table S7 shows the estimated coefficients together with the 95% CI as well as the odds ratio. According to the model, having a power greater than 1 increased the odds to punish free riders in the consecutive punishment stage nearly fivefold (β_5). Higher group contributions decreased these odds slightly. For each additional MU invested by the group, the odds for a powerful group member to punish decreased by 1% (β_6).

Table S7. Use of power regression model.
Dependent variable: punishment behaviour (0 = no punishment or antisocial punishment, 1 = punishment of free riders) clustered by group and participant.

		estimate	odds ratio	95% CI
α_3	intercept (round 3)	-1.03		[-1.78, -0.28]
β_3	round	-0.12	0.89	[-0.18, -0.06]
β_4	group contribution	-0.01	0.99	[-0.02, 0.00]
β_5	power	1.55	4.71	[0.85, 2.23]
β_6	group contribution × power	-0.01	0.99	[-0.02, -0.001]
$\sigma^2_{\alpha_1}$	error-term random intercepts (group level)	0.62		[0.02, 1.17]
$\sigma^2_{\beta_1}$	error-term round slopes (group level)	1.66		[1.19, 2.12]
$\sigma^2_{\alpha_2}$	error-term random intercepts (individual level)	0.09		[0.03, 0.16]
$\sigma^2_{\beta_2}$	error-term round slopes (individual level)	0.13		[0.08, 0.18]
σ^2_y	error-term y	49.97		[0.04, 95.00]
ρ_1	correlation random effects (group level)	-0.30		[-1.00, 0.68]
ρ_2	correlation random effects (individual level)	-0.64		[-0.88, -0.35]

Effect of power on punishment expenses To see whether gaining power influenced not only punishment behavior but also mere punishment expenditure we looked at whether participants spend more MUs on punishment after they received power. Gaining power was weakly correlated with spending more MUs on punishment (r = .15, Figure S14).

To check whether this relation holds controlling for cooperation rates and considering the nested data structure, we fitted a regression model that followed the same random structure described in Equation 7 (however note that here we did not use a logistic but a linear model). The dependent variable coded the change in points used to punish compared to last round. Negative values therefore correspond to how many points were spent less on punishment, positive values correspond to how many punishment points were spent more on punishment compared to last round.

As predictors we used the continuous round predictor, the group contribution of the present

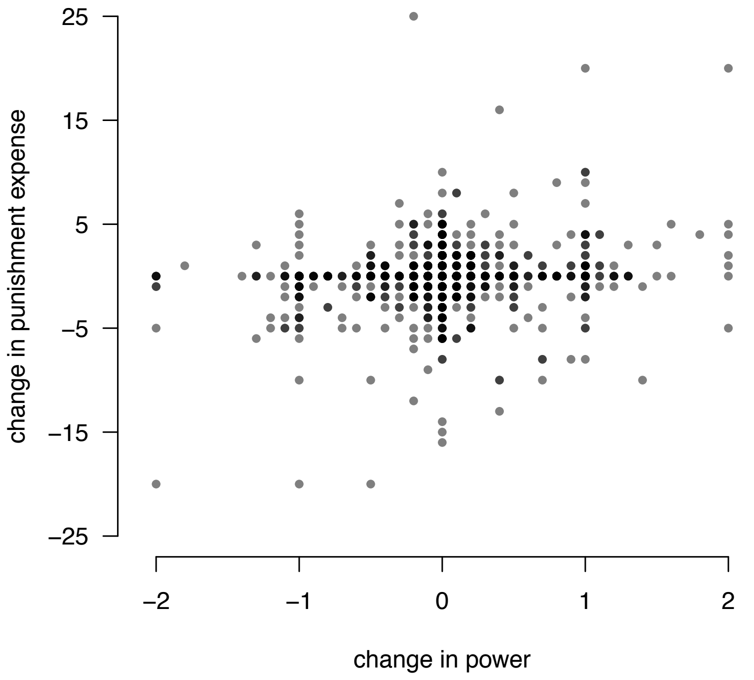

Figure S14. Power and punishment expenses. Relation between change in power from round t-1 and change in points spent on punishment from round t-1.

round, the own contribution of the present round, the absolute power in the present round, the change in power compared to last round, and the difference in power compared to the most powerful member in the group.

Table S8 shows the estimated coefficients together with the 95% CI.

According to the model, group members who contributed more spent more on punishment (β_4), while with higher group contributions fewer points were spent on punishment (β_5). Further, controlling for these effects of contribution on punishment expenses, the more power was gained compared to last round, the more points were spent on punishment and vice versa (β_6).

Effect of power and punishment The regression model for the effect of power and punishment followed the same random structure described in Equation 7 (however note that here we did not use a logistic but a linear model). The dependent variable coded the change in cooperation from previous' round. Since the individual change in cooperation could not exceed 20 and fall below -20, the data were treated as left and right censored.

As predictors we used the continuous round predictor, the reduction in earnings due to punishment in the last round and the change in maximum power from last round, as well as the interaction of change in power and a reduction in earnings due to punishment.

Table S9 shows the estimated coefficients together with the 95% CI. According to the model, contribution decisions were influenced by actual punished, as well as changes in power of the most powerful group member (threat of getting punished). For every MU a participant lost due to getting punished in the last round increased her contribution by 0.3 MUs (β_4). A change in power of 0.1 of the most powerful group member increased contributions by 0.5 MUs (β_5). Additionally, getting punished followed by an increase in power also increased contributions significantly (β_6).

Table S8. Effect of power on punishment expenses regression model.
Dependent variable: change in points spent on punishment from round$_{t-1}$ clustered by group and participant.

		estimate	95% CI
α_3	intercept (change to round 3)	0.03	[-0.32, 0.37]
β_3	round	0.04	[0.02, 0.05]
β_4	own contribution	0.09	[0.07, 0.12]
β_5	group contribution	-0.10	[-0.13, -0.06]
β_6	current power	-0.33	[-0.53, -0.13]
β_7	change in power	0.86	[0.64, 1.07]
β_8	difference in power to most powerful	-0.10	[-0.22, 0.03]
$\sigma^2_{\alpha_1}$	error-term random intercepts (group level)	0.09	[0.00, 0.23]
$\sigma^2_{\beta_1}$	error-term round slopes (group level)	0.01	[0.00, 0.02]
$\sigma^2_{\alpha_2}$	error-term random intercepts (individual level)	0.08	[0.00, 0.21]
$\sigma^2_{\beta_2}$	error-term round slopes (individual level)	0.01	[0.00, 0.02]
σ^2_y	error-term y	2.03	[1.97, 2.09]
ρ_1	correlation random effects (group level)	-0.50	[-1.00, 0.71]
ρ_2	correlation random effects (individual level)	-0.44	[-1.00, 0.73]

Table S9. Effect of power and punishment regression model.
Dependent variable: change in cooperation from round$_{t-1}$ clustered by group and participant.

		estimate	95% CI
α_3	intercept (change to round 3)	-0.12	[-0.49, 0.25]
β_3	round	-0.03	[-0.08, 0.01]
β_4	earnings reduction (from punishment in round $t-1$)	0.31	[0.25, 0.37]
β_5	change in maximum power (from round $t-1$)	4.76	[3.06, 6.48]
β_6	earnings reduction × change in power	0.54	[0.08, 0.99]
$\sigma^2_{\alpha_1}$	error-term random intercepts (group level)	0.46	[0.00, 0.89]
$\sigma^2_{\beta_1}$	error-term round slopes (group level)	0.11	[0.00, 0.28]
$\sigma^2_{\alpha_2}$	error-term random intercepts (individual level)	0.07	[0.01, 0.12]
$\sigma^2_{\beta_2}$	error-term round slopes (individual level)	0.01	[0.00, 0.03]
σ^2_y	error-term y	3.73	[3.62, 3.84]
ρ_1	correlation random effects (group level)	-0.73	[-1.00, 0.23]
ρ_2	correlation random effects (individual level)	-0.27	[-1.00, 0.83]

Getting punished By giving away power in the power transfer game, group members can make themselves rather inefficient punishers, thereby 'creating an excuse' to not engage in costly punishment. They also reveal themselves as second-order free riders, those willing to cooperate but not willing to spend points on sanctioning free riders. This 'shying away from responsibility' and trying to second-order free ride on powerful group members could however motivate others to punish such a behavior.

We therefore analysed what behavior increased the likelihood of getting punished in the endogenous condition. In particular, we tested whether second-order free riding and/or transfering power to another group member increased the likelihood of getting punished in two separate analyses.

First, we looked at first and second round behavior in which power transfer was not possible yet and classified participants into three types: Those who contributed equal or above group average and punished equal or above group average (initial punishing cooperators), those who contributed equal or above average but punished below average (initial second-order free riders), and those who both contributed and punished below group average (initial first-order free riders). As mentioned in the paper, participants who initially contributed above group average and punished above group average received significantly more power over the course of the experiment than first- and second-order free riders. Figure S15 shows the average punishment points received over rounds 3 to 20. The behavior in the first two rounds significantly affected the amount of punishment received over the whole experiment (Kruskal-Wallis Test, $\chi^2(2) = 13.1$, P < .01). Post-hoc Dunn tests, using Bonferroni correction for multiple comparisons, revealed that the amount of punishment received did not significantly differ between initial punishing cooperators and second-order free riders (Dunn Test, $z(2)$ = -0.67, P = .75). However, initial first-order free riders got punished significantly more, on average, than initial punishing cooperators (Dunn Test, $z(2) = 2.78$, P < .01) and second-order free riders (Dunn Test, $z(2) = 3.14$, P < .01).

Second, we looked at round to round behavior in a regression analysis. The regression model followed the same random structure described in Equation 7. The dependent variable coded whether a participant was punished or not (0 indicated no punishment received and 1 indicated getting punished) in a certain round. As predictors we used the continuous round predictor, the amount of points spent on punishment in the previous round compared to group average as a dummy predictor coding second-order free riding (0 = punishment expense equal or above group average, 1 = punishment expense below group average), a dummy predictor indicating (first-order) free riding (0 = equal or above average contribution, 1 = below average contribution), the amount of power transferred, and the current power status in the group (amount of power in the present round).

Table S10 shows the estimated coefficients together with the 95% CI as well as the odds ratio. According to the model, contributing less than group average increased the odds of getting punished in the consecutive punishment stage twelvefold (β_4). Punishing less than group average only increased the odds of getting punished 1.6 fold (β_5). Giving up and transferring power did not significantly increase or decrease the odds of getting punished (β_6). Thus, punishment was mainly aimed at free riders, those who contributed less to the public good and was not statistically related to transferring power.

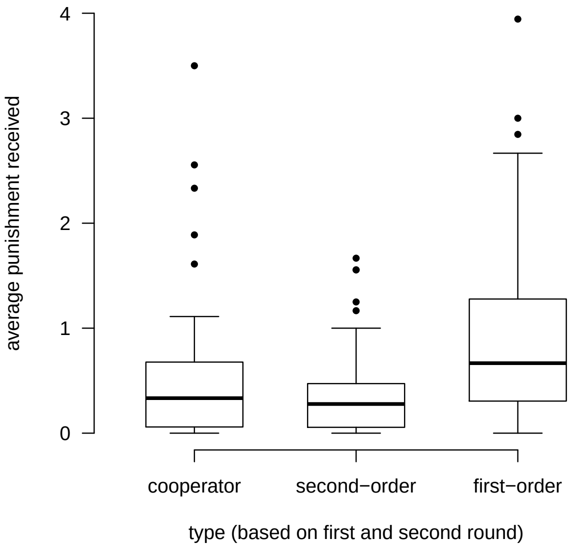

Figure S15. Initial behavior and received punishment. Average punishment received for different cooperation types defined based on first round and second round behavior. Cooperator: Participants who contributed equal/above group average to the public good in the first round and punished equal/above group average, second-order: Participants who contributed equal/above group average but punished below group average, first-order: Participants who contributed below average and punished below average.

Table S10. Getting punished regression model.
Dependent variable: getting punished clustered by group and participant.

		estimate	odds ratio	95% CI
α_3	intercept (change to round 3)	-1.91		[-2.78, -1.04]
β_3	round	-0.08	0.92	[-0.13, -0.03]
β_4	cooperation type	2.49	12.06	[2.18, 2.81]
β_5	punishment type$_{t-1}$	0.46	1.58	[0.19, 0.73]
β_6	amount of power transferred	-0.44	0.64	[-1.02, 0.15]
β_7	current power	-0.28	0.76	[-0.66, 0.10]
$\sigma^2_{\alpha_1}$	error-term random intercepts (group level)	1.18		[0.72, 1.68]
$\sigma^2_{\beta_1}$	error-term round slopes (group level)	0.10		[0.05, 0.15]
$\sigma^2_{\alpha_2}$	error-term random intercepts (individual level)	0.59		[0.01, 1.04]
$\sigma^2_{\beta_2}$	error-term round slopes (individual level)	0.04		[0.00, 0.09]
σ^2_y	error-term y	50.02		[1.56, 96.59]
ρ_1	correlation random effects (group level)	-0.59		[-0.91, -0.19]
ρ_2	correlation random effects (individual level)	-0.44		[-1.00, 0.76]

Individual level analysis for the exogenous condition

To analyse how gaining power and a change in maximum power affected individual decisions in the exogenous condition in which power transfers were not possible, we fitted the 'use of power' and the 'effect of power and punishment' models described above also to the data of the exogenous condition groups. The regression models followed the same structure as described above for the endogenous condition.

Table S11 shows the estimated coefficients together with the 95% CI as well as the odds ratio for the use of power regression model. Table S12 shows the estimated coefficients together with the 95% CI for the effect of power and punishment regression model.

Like in the endogenous condition, having a power greater than 1 increased the odds to punish free riders in the consecutive punishment stage (β_5, Table S11). Individual contributions in turn increased after experiencing a reduction in earnings due to punishment, similarly to the endogenous condition (β_4, Table S12). However, contrary to the endogenous condition, a change in maximum power from last round (threat of punishment) did not affect contribution decisions significantly (β_5, Table S12).

Thus, the difference in cooperation we observe over rounds between the endogenous and exogenous condition can not be attributed to a lower willingness to punish free riders after receiving power or a lower effect of punishment on cooperation. Instead, observing an increase in power centralisation already increased cooperation of group members in the endogenous condition, while power centralisation did not affect cooperation of group members in the exogenous condition.

Table S11. Use of power regression model for the exogenous condition. Dependent variable: punishment behaviour (0 = no punishment or antisocial punishment, 1 = punishment of free riders) clustered by group and participant.

		estimate	odds ratio	95% CI
α_3	intercept (round 3)	-2.57		[-3.33, -1.78]
β_3	round	-0.01	0.99	[-0.05, 0.03]
β_4	group contribution	0.00	1.00	[-0.01, 0.02]
β_5	power	1.69	5.42	[1.03, 2.34]
β_6	group contribution × power	-0.01	0.99	[-0.02, 0.00]
$\sigma^2_{\alpha_1}$	error-term random intercepts (group level)	0.79		[0.30, 1.30]
$\sigma^2_{\beta_1}$	error-term round slopes (group level)	1.67		[1.26, 2.07]
$\sigma^2_{\alpha_2}$	error-term random intercepts (individual level)	0.06		[0.01, 0.10]
$\sigma^2_{\beta_2}$	error-term round slopes (individual level)	0.09		[0.05, 0.13]
σ^2_y	error-term y	50.01		[0.18, 95.02]
ρ_1	correlation random effects (group level)	-0.18		[-0.84, 0.80]
ρ_2	correlation random effects (individual level)	-0.79		[-0.97, -0.59]

Table S12. Effect of power and punishment regression model for the exogenous condition. Dependent variable: change in cooperation from round$_{t-1}$ clustered by group and participant.

		estimate	95% CI
α_3	intercept (change to round 3)	-0.25	[-0.73 0.22]
β_3	round	-0.03	[-0.08 0.02]
β_4	earnings reduction (from punishment in round $t-1$)	0.38	[0.30 0.46]
β_5	change in maximum power (from round $t-1$)	-0.13	[-0.54 0.29]
β_6	earnings reduction $\times$ change in power	0.19	[0.08 0.30]
$\sigma^2_{\alpha_1}$	error-term random intercepts (group level)	0.15	[0.00, 0.38]
$\sigma^2_{\beta_1}$	error-term round slopes (group level)	0.23	[0.00, 0.66]
$\sigma^2_{\alpha_2}$	error-term random intercepts (individual level)	0.02	[0.00, 0.04]
$\sigma^2_{\beta_2}$	error-term round slopes (individual level)	0.03	[0.00, 0.08]
σ^2_y	error-term y	4.56	[4.42, 4.69]
ρ_1	correlation random effects (group level)	-0.26	[-1.00, 0.83]
ρ_2	correlation random effects (individual level)	-0.25	[-1.00, 0.82]

Other remarks

Power and cooperation In Figure 3b of the manuscript, we reported the correlation of maximum power and average cooperation across rounds for each group. For the calculation of these correlations we omitted round 20 from each group. This was done because of the sharp drop of cooperation in the last round (known as the endgame effect). As can be seen in Figure S16, with increased maximum power, mean contribution increased. Round 20 can be identified as an outlier.

Including round 20 in the reported analysis did not change the statistical inferences reported in the manuscript. Quantitatively, the average correlation dropped from r = 0.24 to r = .21 for the exogenous condition groups.

Power and earnings As reported in the manuscript, receiving power was correlated with lower earnings compared to the other group members. Figure S17 shows how earnings decreased, the more power a participant received in the experiment.

First and second round types In the manuscript, when examining the connection between behaviour in the first two rounds, and later power status, we omitted an analysis of antisocial punishers, because antisocial punishment was very rare. The distribution of initial punishment behaviour was as follows: 75 participants decided not to punish in the second round, 49 participants punished primarily free riders, and only 11 participants punished antisocially.

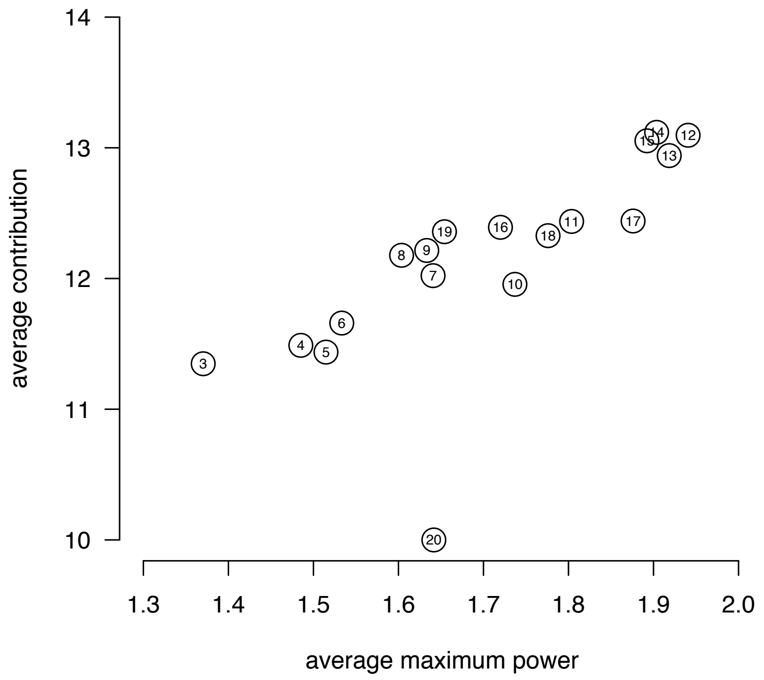

Figure S16. Average cooperation and average maximum power in each round of the exogenous condition. The numbers in each circle indicate the round.

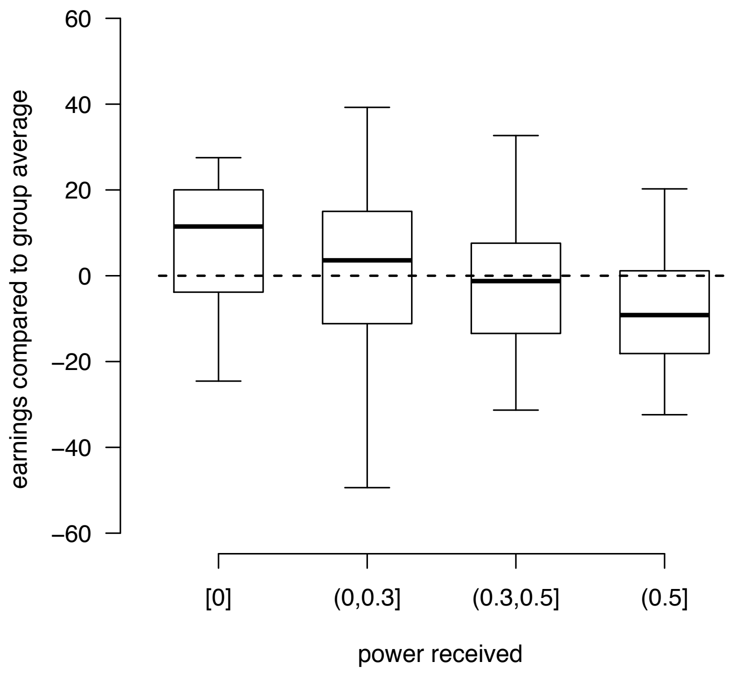

Figure S17. Earnings and power. Average earnings of group members compared to the group average for different average amounts of power received over the experiment. Horizontal bars depict the median.
